# Transcriptome and metabolite profiling analyses provide insight into volatile compounds of the apple cultivar ‘Ruixue’ and its parents during fruit development

**DOI:** 10.1186/s12870-021-03032-3

**Published:** 2021-05-24

**Authors:** Xiaojie Liu, Nini Hao, Ruifang Feng, Zhipeng Meng, Yanan Li, Zhengyang Zhao

**Affiliations:** College of Horticulture, Northwest A&F University, Yangling, 712100 Shaanxi China

**Keywords:** Volatile aroma compounds, ‘Ruixue’ (*Malus* × *domestica* Borkh.), Fruit flavour, Gas chromatography-mass spectrometry (GC–MS), Gene expression

## Abstract

**Background:**

Aroma is one the most crucial inherent quality attributes of fruit. ‘Ruixue’ apples were selected from a cross between ‘Pink Lady’ and ‘Fuji’, a later ripening yellow new cultivar. However, there is little known about the content and composition of aroma compounds in ‘Ruixue’ apples or the genetic characters of ‘Ruixue’ and its parents. In addition, the metabolic pathways for biosynthesis of aroma volatiles and aroma-related genes remain poorly understood.

**Results:**

Volatile aroma compounds were putatively identified using gas chromatography-mass spectrometry (GC–MS). Our results show that the profile of volatile compounds changes with ripening. Aldehydes were the dominant volatile compounds in early fruit development, with alcohols and esters increasing dramatically during maturation. On the basis of a heatmap dendrogram, these aroma compounds clustered into seven groups. In ripe fruit, esters and terpenoids were the main aroma volatiles in ripening fruit of ‘Pink Lady’ and ‘Fuji’ apples, and they included butyl 2-methylbutanoate; propanoic acid, hexyl ester; propanoic acid, hexyl ester; hexanoic acid, hexyl ester; acetic acid, hexyl ester and (Z, E)-α-farnesene. Interestingly, aldehydes and terpenoids were the dominant volatile aroma compounds in ripening fruit of ‘Ruixue’, and they mainly included hexanal; 2-hexenal; octanal; (E)-2-octenal; nonanal and (Z, E)-α-farnesene. By comparing the transcriptome profiles of ‘Ruixue’ and its parents fruits during development, we identified a large number of aroma-related genes related to the fatty acid, isoleucine and sesquiterpenoid metabolism pathways and transcription factors that may volatile regulate biosynthesis.

**Conclusions:**

Our initial study facilitates a better understanding of the volatile compounds that affect fruit flavour as well as the mechanisms underlying differences in flavour between ‘Ruixue’ and its parents.

**Supplementary Information:**

The online version contains supplementary material available at 10.1186/s12870-021-03032-3.

## Background

The quality of fruit is mainly determined by appearance, texture, flavour and nutritional properties [1]. Fruit flavour, one the most crucial inherent quality attributes, is determined by sugars, organic acids and aroma [2]. Fruit has a strong aroma that can directly reflect its flavour characteristics, a crucial factor affecting consumer acceptance and market competitiveness [3]. The approximately 2000 volatile compounds associated with aromas in fruits are highly complex and diverse. To date, approximately 350 volatile compounds have been identified and quantified and in apple [2], mainly identifiable as esters, alcohols, aldehydes, phenols, ethers, terpenes, ketones and some sulphur compounds. Among these only a small number contribute prominently to fruit aroma [2, 4].

Volatile aroma compounds are produced through four primary metabolic pathways including the fatty acid, isoleucine, mevalonate and phenylpropanoid pathways [5]. Aldehydes, alcohols and esters (straight-chain) are synthesized from fatty acid metabolic pathways through β-oxidation and lipoxygenase activity (LOX). The lipoxygenase (LOX) pathway involves lipoxygenase (LOX), hydroperoxidelyase (HPL), alcohol dehydrogenase (ADH) and alcohol acyl transferases (AATs) [6]. Lipoxygenase (LOX) is the initial step in ester (straight-chain) biosynthesis during fatty acid degradation (linoleic and linolenic). In apples a total of 23 potential functional lipoxygenase (LOX) genes have been putatively identified [7]. Gene expression analysis and QTL mapping experiments indicate that *MdLOX1a* and *MdLOX5e* are involved in fruit aroma volatile production in a molecular breeding approach [7]. Alcohol acyl transferases (AAT) are involved in the final steps and in rate limiting ester biosynthesis [8]. Alcohol acyl transferase 1 (*MpAAT1*) has been isolated from ‘Royal Gala’ apples, catalysing the synthesis of the primary esters [9]. *MpAAT1* has been co-located with ester QTLs (LG2) and transgenic apples (AAT1 knockdown lines) exhibiting a significant decrease in total ester concentrations, especially in 2-methylbutyl acetate and butyl propanoate [10]. In addition, alcohol acyl transferase 2 (*MdAAT2*) is highly expressed and significantly correlated with ester production in ‘Granny Smith’ apples [11]. It is reported that there is differential expression of AAT gene family members in different apple cultivars [12]. Distinct plant genotype and substrate specificity of AAT enzymes is a prominent factor contributing to cultivar differences in production of ester volatiles [6, 13]. Terpenes are produced by the mevalonate (MVA) pathway in the cytoplasm [5]. Terpene synthases (TPS) are the key enzymes in synthesis of volatile terpenes. In apple, a total of 55 putative apple TPS family members were putatively identified, and functional analysis found that only 10 TPS genes are predicted to be functional, synthesizing most of the terpene volatiles produced in ‘Royal Gala’ apples such as D-germacrene, linalool and a-pinene [14]. It is worth noting that α-farnesene synthase (MdAFS1) of TPS enzymes and its oxidative product have been putatively identified as key factors causing superficial scald, a postharvest physiological disorder [15].

The differences in content and composition of aroma compounds among varieties produce different flavours in fruit. In ‘Golden Delicious’ apples, a total of 65 volatile aroma compounds(free and bound forms) have been putatively identified and quantified. Among these, esters and alcohols accounted for greater than 90% of total volatiles, butylacetate (156.70ug/kg), 2-methyl-1-propanol (126.72ug/kg) and n-butanol (473.28ug/kg) were the primary compounds [16]. In ‘Fuji’ apples, a total of 64 volatile aroma compounds have been putatively identified and quantified. Among these, alcohols have been putatively identified as dominant compounds, and the second is esters compounds, with 2-methyl-1-butanol (368.05ug/kg), n-butanol (776.40ug/kg), and butylacetate (71.52ug/kg) the most abundant [16]. A total of 63 volatile compounds were putatively detected in ‘Pink Lady’ apples, the main volatile compounds were n-butanol followed by 2-methyl-1-butanol, 1-hexanol, butyl acetate [16, 17]. Recently, the characteristic aroma of ‘Honeycrisp’ apples was putatively identified and quantified using gas chromatography–olfactometry (GC-O) and aroma extract dilution analysis (AEDA) [4]. Hexyl-methylbutyrate, hexyl 2-methylbutyrate, α-farnesene, and (E)-2-hexenal were dominant contributors to the aroma of ‘Honeycrisp’ apples.

‘Ruixue’ apples were selected from a cross between ‘Pink Lady’ and ‘Fuji’, a later ripening yellow apple cultivar. A preliminary study of the quality of 'Ruixue' fruit in our laboratory, sensory analysis shows that its flesh is crisp, juicy, and sweet, having a distinct aromatic flavour compared to its parents [18]. However, there is little known about the content and composition of aroma compounds in ‘Ruixue’ apples or the genetic characters of ‘Ruixue’ and its parents. In addition, the metabolic pathways for biosynthesis of aroma volatiles and aroma-related genes remain poorly understood. In this study, volatile aroma compounds were putatively identified and quantified for ‘Ruixue’, ‘Pink Lady’ and ‘Fuji’ apples during fruit development using gas chromatography-mass spectrometry (GC–MS) and compared between the three cultivars. RNA sequencing (RNA-seq) transcriptome data and quantitative reverse transcriptase-PCR were used to identify candidate structural genes and potential transactional factors associated with aroma synthesis by the fatty acid, isoleucine and sesquiterpenoid metabolism pathways in ‘Ruixue’, ‘Pink Lady’ and ‘Fuji’ apples during fruit development. This study aims to clarify aroma profiles and further reveal the mechanisms underlying differential volatile aroma compounds between ‘Ruixue’ and its parents that affect the flavour of these three cultivars.

## Results

### Physiological characteristics of ‘Ruixue’ and its parents apples during fruit development

**Fig. 1:**
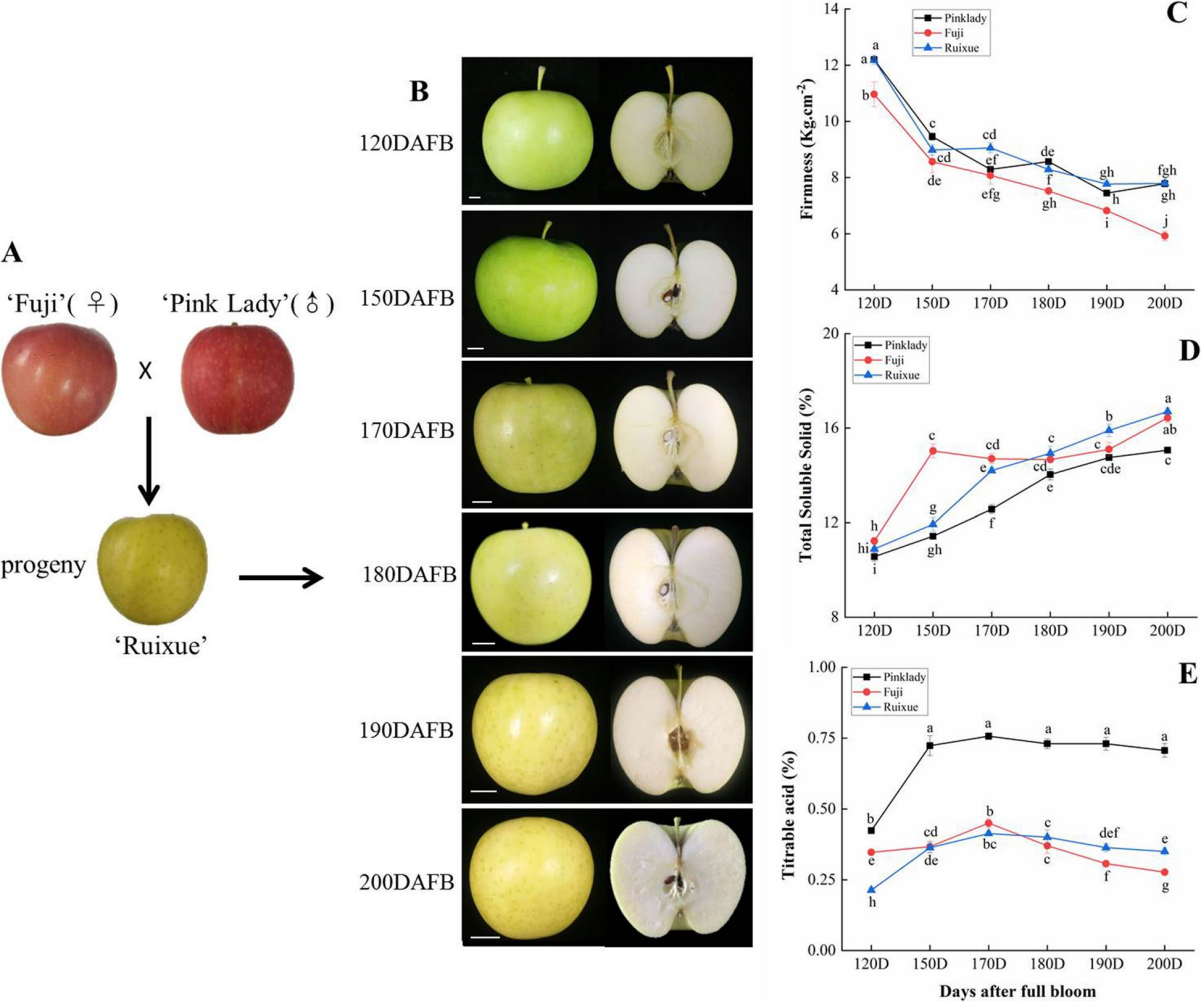
Physiological characters of‘Ruixue’ and its parents apples during fruit development. **a** Appearance of Ruixue’ and its parents apples. **b** Appearance of‘Ruixue’ fruit at various development stages: 120 DAFB, 150 DAFB, 170 DAFB, 180 DAFB, 190 DAFB and 200 DAFB. **c** Fruit firmness of ‘Ruixue’ and its parents apples during fruit development. **d** Total soluble solids (TSS) of ‘Ruixue’ and its parents apples during fruit development. **e** Titratable acidity (TA) of ‘Ruixue’ and its parents apples during fruit development. DAFB: days after full bloom. Error bars show ± SE from three biological replicates. Significant differences are showed with different letters above the bars (*P* < 0.05, Duncan’s new multiple range test). Bars = 1 cm

The ‘Ruixue’ apple, a yellow cultivar, was selected from a cross between ‘Pink Lady’ and ‘Fuji’ (Fig. 1a). A total of six developmental stages were collected from 120 DAFB to the ripened stages. The colour of the ‘Ruixue’ apple peel changed from green to yellow and the colour of the pulp from green to milky white (Fig. 1b). Fruit firmness, total soluble solids and titrable acidity are critical factors for fruit quality. Fruit firmness of the three cultivars showed a dramatically decline before 150 DAFB (Fig. 1c), and then declined slowly during latter development. There were no significant differences in fruit firmness between ‘Ruixue’ and ‘Pink Lady’ at 200 DAFB (maturation stage). However, the fruit firmness of ‘Ruixue’ was greater, approximately 1.3-fold that of ‘Fuji’ at 200 DAFB (maturation stage). The total soluble solids of the three cultivars showed a significant increase during fruit development. (Fig. 1d). The total soluble solids of ‘Ruixue’ were greater than that of ‘Pink Lady’ from 170 DAFB until 200 DAFB, and also significantly lower than of ‘Fuji’ at 150 DAFB. The titrable acidity of the three cultivars showed a first increase before 170 DAFB, and then maintained at a steady level in the ‘Pink Lady’ and ‘Ruixue’ after 170 DAFB (Fig. 1e). However, the titrable acidity of ‘Fuji’ apple declined after 170 DAFB. The titrable acidity of ‘Ruixue’ was significantly lower than that of ‘Pink Lady’ during fruit development. There were no differences in the titrable acidity between ‘Ruixue’ and ‘Fuji’ apple after 120 DAFB. Generally, ‘Ruixue’ apple fruit in fruit firmness, total soluble solids and titrable acidity have differences from its parents apples during fruit development.

### Volatiles profile of ‘Ruixue’ and its parents apples during fruit development

The volatile compounds and relative changes in content of ‘Ruixue’ and its parents apples were putatively identified during different fruit developmental stages. A total of 54 volatiles were monitored from ‘Ruixue’, ‘Fuji’ and ‘Pink Lady’ during fruit development (Supplementary Table S1), including 35 esters (the most abundant volatiles), 9 aldehydes, 5 alcohols, 4 terpenoids and 1 acid. ‘Ruixue’ contained 40 volatiles: 25 esters, 8 aldehydes, 3 alcohols, 3 terpenoids and 1 acid. ‘Fuji’ contained 46 aroma volatiles: 29 esters, 9 aldehydes, 4 alcohols, 3 terpenoids and 1 acid. ‘Pink Lady’ contained only 32 volatiles: 22 esters, 4 aldehydes, 3 alcohols, 2 terpenoids and 1 acid.

**Fig. 2:**
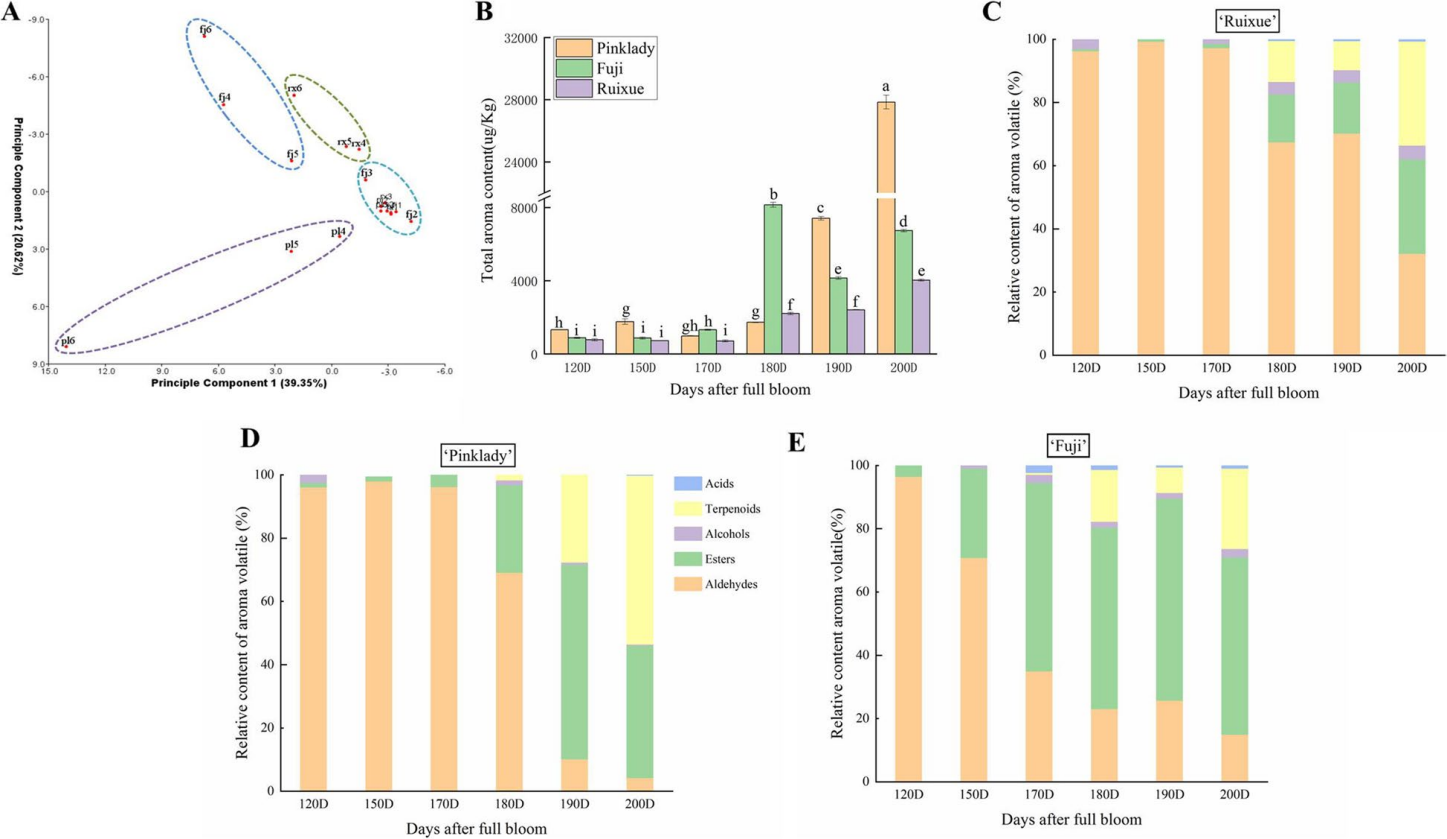
Volatile profile of‘Ruixue’ and its parents apples during fruit development. **a** Principal component analysis (PCA) of volatile compounds in ‘Ruixue’ and its parents apples during fruit development. **b** Total aroma content (μg/kg) during fruit development. **c** Relative content of main volatile components in ‘Ruixue’ during fruit development. **d** Relative content of main volatile components in ‘Pink Lady’ during fruit development. **e** Relative content of main aroma components in ‘Fuji’ during fruit development. DAFB: days after full bloom. Error bars show ± SE from three biological replicates. Significant differences are showed with different letters above the bars (*P* < 0.05, Duncan’s new multiple range test)

A principal component analysis (PCA) was performed to characterize apple cultivar ‘Ruixue’ and its parents with respect to their volatile profiles during fruit development. In the PCA plot (Fig. 2a), the first two components could explain 59.97% of the variability in volatile profiles, 39.35% (principal component 1, PC1) and 20.62% (principal component 2, PC2). There was a no difference in ‘Ruixue’ and its parents before170 DAFB, however, in later stages of development (170–200 DAFB), showed quite different characters in ‘Ruixue’ and its parents. The total volatile contents of ‘Ruixue’ and its parents apples increased slowly from 120 to 150 DAFB then increased rapidly from 150 to 200 DAFB. In contrast to ‘Fuji’ (180DAFB), the total volatiles content of ‘Ruixue’ and ‘Pink Lady’ reached maximum levels at 200 DAFB (Fig. 2b). The relative content of volatiles (esters, aldehydes, alcohols, terpenoids and acids) during fruit development exhibited dynamic change (Fig. 2c, d and e). For ‘Ruixue’, aldehydes were the main volatiles and account for more than 96% of total volatiles prior to 170 DFAB (immature fruit), then declined to a minimum (32.19%) at 200 DAFB (mature fruit). Esters began to accumulate after 170 DAFB, reaching a maximum (29.91%) at 200 DAFB. Similarly the relative terpenoid content exhibited a significant increase after 170 DAFB, reaching its maximum (32.91%) at 200 DAFB, becoming the most prominent aroma volatile group (Fig. 2c). Few alcohols and acids were putatively detected during fruit development, and the results for ‘Pink Lady’ and ‘Fuji’ were similar. For ‘Pink Lady’, aldehydes were the predominant volatiles and account for more than 90% of total volatile content before 170 DFAB (immature fruit) then rapidly decreased to a minimum (4.28%) at 200 DAFB. Conversely, terpenoids began to accumulate after 170 DAFB, reaching a maximum (53.37%) at 200 DAFB (Fig. 2d). For ‘Fuji’, the relative content of aldehydes continued to decrease during fruit development and reached a minimum (14.95%) at 200 DAFB; however, the relative content of esters increased to a maximum level (63.92%) at 190 DAFB and then declined (Fig. 2e).

**Fig. 3:**
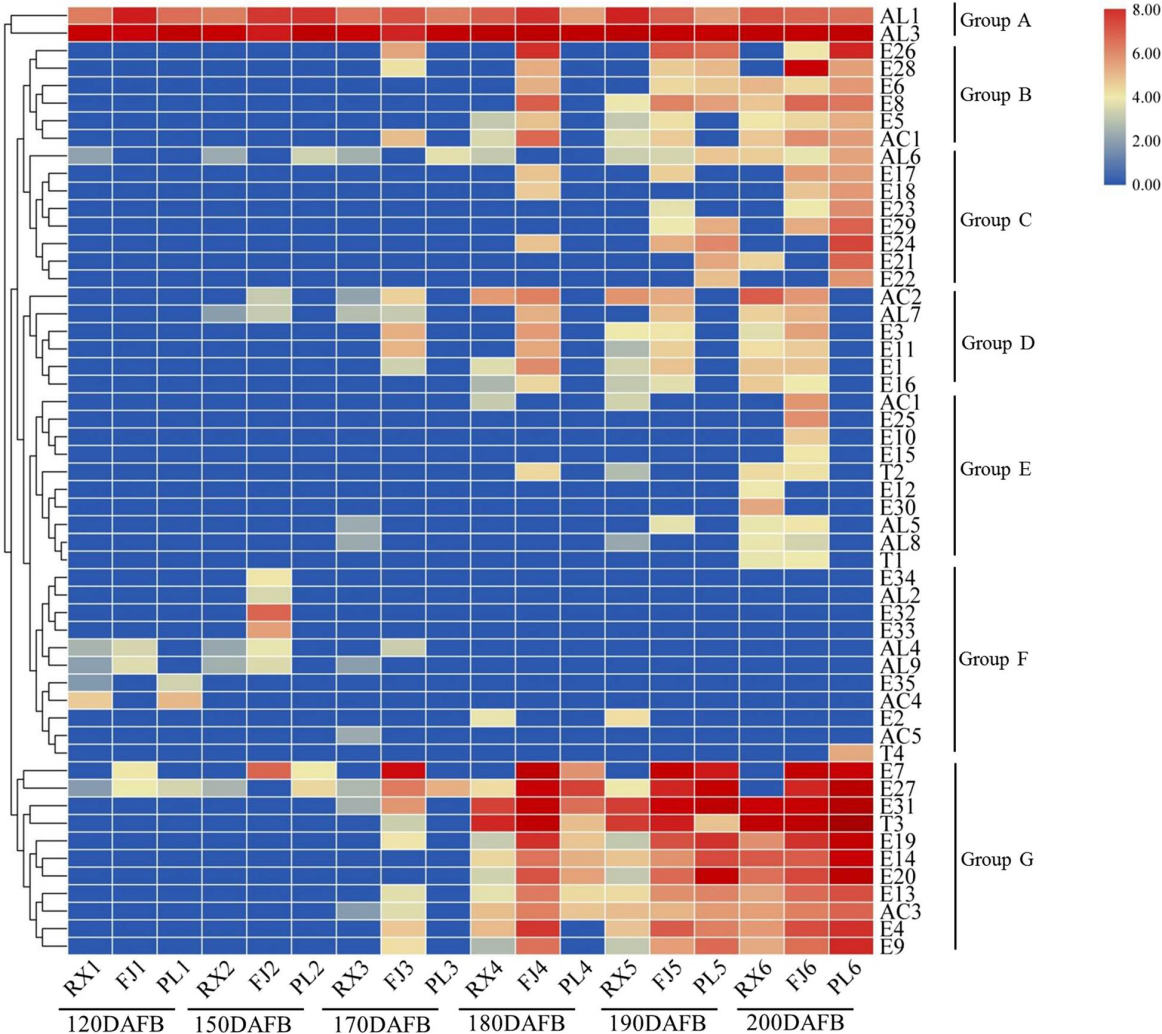
Heatmap with hierarchical clustering of volatile content (μg/kg) in ‘Ruixue’ and its parents apples during fruit development. DAFB: days after full bloom. “F1, F2 F3, F4, F5 and F6” respectively represents at 120, 150, 170, 180, 190 and 200 DAFB(days after full bloom) of ‘Fuji’; “P1, P2, P3, P4, P5 and P6” respectively represents1 at 120, 150, 170, 180, 190 and 200 DAFB(days after full bloom) of ‘Pink Lady’; “X1, X2, X3, X4, X5 and X6” respectively represents at 120,150,170,180,190 and 200 DAFB(days after full bloom) of ‘Ruixue’

To visualise the apple fruit aroma volatiles profile, volatiles of ‘Ruixue’, ‘Fuji’ and ‘Pink Lady’ during fruit development were analysed for hierarchical clustering. As shown in Fig. 3, volatiles putatively detected in ‘Ruixue’, ‘Fuji’ and ‘Pink Lady’ during fruit development clustered into seven groups (A, B, C, D, E, F and G) in the Heatmap dendrogram. Group A, consisting of two compounds AL1 (Hexanal) and AL3 (2-Hexenal), showed an increasing trend and maintained high content level in three cultivars during fruit development. There was a difference in content of AL1 and AL3 of ‘Ruixue’ compared to its parents. Group B, consisting of six compounds E26 (Acetic acid, butyl ester), E28 (Acetic acid, pentyl ester), E6 (Hexanoic acid, 2-methylbutyl ester), E8 (Propanoic acid, butyl ester), E5 (Butanoic acid, 2-methyl-, pentyl ester) and AC1 (Butanoic acid, 2-methyl-), showed an increasing trend in three cultivars after 180DAFB, and was more abundant in ‘Pink Lady’ fruit. Group C, consisting of eight compounds AL6 (2-Heptenal, (Z)-), E17 (Heptanoic acid, butyl ester), E18 (Hexanoic acid, propyl ester), E23 (2-Methylbutyl octanoate), E29 (Propanoic acid, 2-methyl-, hexyl ester), E24 (Butyl caprylate), E21 (Hexanoic acid, pentyl ester) and E22 (Octanoic acid, hexyl ester) showed low content in three cultivars during fruit development, and content was lowest in ‘Ruixue’ compared to its parents. Group D, consisting of six compounds AC2 (1-Butanol, 2-methyl-), AL7 (Nonanal), E3 (Butanoic acid, 2-methyl-, propyl ester), E11 (Propanoic acid, pentyl ester), E1 (2-Methylbutyl 2-methylbutyrate) and E16 (Butanoic acid, 2-methylbutyl ester), showed an increasing trend in three cultivars during fruit development, and was more abundant in ‘Ruixue’ fruit. Group E, consisting of ten compounds AC1 (1-Butanol), E25 (Acetic acid propyl ester), E10 (Propanoic acid, propyl ester), E15 (Butanoic acid, ethyl ester), T2 (5-Hepten-2-one, 6-methyl-), E12 (Butanoic acid, propyl ester), E30 (Propanoic acid, 2-methyl-, pentyl ester), AL5 (Octanal), AL8 (2-Octenal, (E)-) and T1 (1-Octen-3-one), showed low content in three cultivars during fruit development, and was more abundant in ‘Ruixue’ fruit. Group F, consisting of eleven compounds E34 (2-Hexen-1-ol, acetate, (Z)-), AL2 (4-Pentenal, 2-methyl-), E32 (2-Methylbut-2-en-1-yl acetate), E33 (3-Hexen-1-ol, acetate, (E)-), AL4 (3-Hexenal), AL9 (2,4-Hexadienal, (E,E)-), E35 (Methyl salicylate), AC5((5-Methyltetrahydro-2-Furanyl)Methanol) and T4 (Isocaryophillene), showed an decreasing trend in three cultivars during fruit development. Group G, consisting of eleven compounds E7 (1-Butanol, 2-methyl-, acetate), E27 (Acetic acid, hexyl ester), E31 (Butanoic acid, 2-methyl-, hexyl ester), T3 ((Z,E)-α-Farnesene), E19 (Hexanoic acid, butyl ester), E14 (Butanoic acid, hexyl ester), E20 (Hexanoic acid, hexyl ester), E13 (Butanoic acid, butyl ester), AC3(1-Hexanol), E4 (Butyl 2-methylbutanoate) and E9 (Propanoic acid, hexyl ester), showed an increasing trend and maintained high content level in three cultivars during fruit development.

In 200 DAFB (fruit mature period), a total of 29 volatiles were putatively detected in ‘Ruixue’ apple fruit, including 17 esters, 6 aldehydes, 2 alcohols, 3 terpenoids and 1 acid. For ‘Pink Lady’ a total of 28 aroma volatiles were putatively identified, including 21 esters, 3 aldehydes, 1 alcohol, 2 terpenoids and 1 acid. In ‘Fuji’ apples, 31 aroma volatiles were putatively identified, including 22 esters, 5 aldehydes, 2 alcohols, 1 terpenoid and 1 acid. Interestingly, in ‘Ruixue’ apples, aldehydes (hexanal, 2-hexenal and (E)-2-octenal), esters (butanoic acid, propyl ester, butanoic acid, 2-methylbutyl ester, propanoic acid, 2-methyl-, pentyl ester), alcohols (alcohol) and terpenoids (5-hepten-2-one, 6-methyl-) were significantly higher than in ‘Fuji’ and ‘Pink Lady’. Conversely, esters (butyl 2-methylbutanoate, propanoic acid, hexyl ester, butanoic acid, butyl ester, heptanoic acid, butyl ester, hexanoic acid, butyl ester and hexanoic acid, and hexyl ester) were significantly lower than in ‘Fuji’ and ‘Pink Lady’. In summary, there were differences in aroma volatile composition and content in different apple cultivars.

### Transcriptome analysis of ‘Ruixue’ and its parents apples during fruit development

In order to further explore the molecular mechanisms underlying differences in flavour between ‘Ruixue’ and its parents, RNA-sequencing (RNA-Seq) was utilized to obtain genome-wide gene expression profiles during fruit development with same samples used for volatile compound analysis. A total of 54 samples (three cultivars × six developmental stages × three biological replicates) were subjected to RNA-seq analysis in order to identify differentially expressed genes related to volatile compound biosynthesis and transcriptional regulation responsible for the diverse flavours exhibited by the three cultivars. After filtering, a total of 1,073,298,706 clean reads were obtained from ‘Ruixue’fruit, 979,721,018 and 1,015,631,116 clean reads were produced from ‘Fuji’ and ‘Pink Lady’ fruit, respectively. A total of 460.76 GB nucleotides were obtained with an average GC content of 47.28%. Q30 percentage (error rates lower than 0.1%) was over 90% (Supplementary Table S2).

**Fig. 4:**
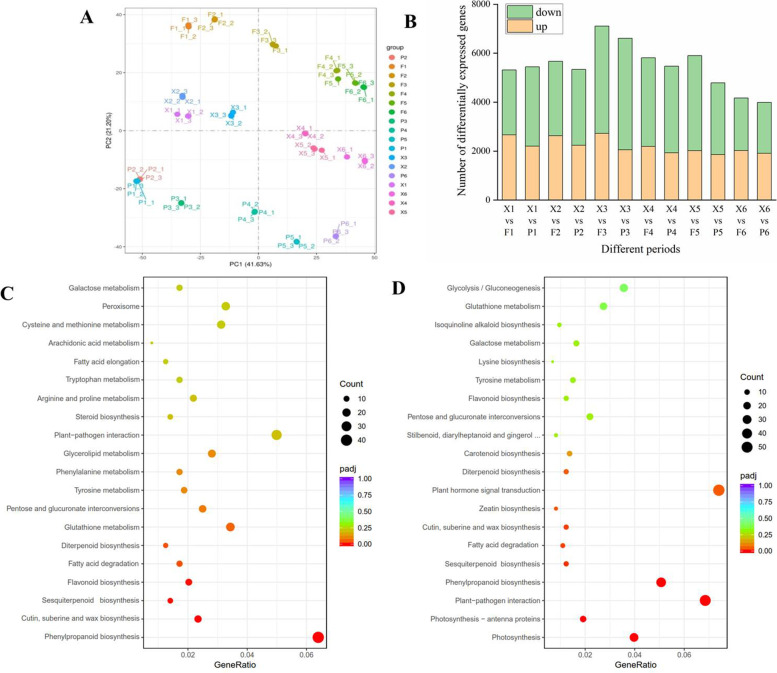
Summary analysis of transcriptomes. **a** Principal component analysis (PCA) of transcriptome of 54 samples from ‘Ruixue’ and its parents apples during fruit development. **b** Differentially expressed genes (DEGs) of ‘Ruixue’ and its parents apples during fruit development. **c** KEGG pathway analysis of‘Ruixue’ and ‘Fuji’ at 170 DAFB. **d** KEGG pathway analysis of‘Ruixue’ and ‘Pink Lady’ at 170 DAFB. “F1, F2 F3, F4, F5 and F6” respectively represents at 120, 150, 170, 180, 190 and 200 DAFB(days after full bloom) of ‘Fuji’; “P1, P2, P3, P4, P5 and P6” respectively represents1 at 120, 150, 170, 180, 190 and 200 DAFB(days after full bloom) of ‘Pink Lady’; “X1, X2, X3, X4, X5 and X6” respectively represents at 120,150,170,180,190 and 200 DAFB(days after full bloom) of‘Ruixue’

Principal component analysis was based on the transcriptome profiles from 54 samples. The first two principal components explain 41.63% (PC1) and 21.20% (PC2) of the variance among the samples. Similarities and differences among the apple transcriptomes were mostly driven by fruit developmental stage. Additionally, ‘Ruixue’, ‘Fuji’ and ‘Pink Lady’ cultivars separated on PC2 (Fig. 4a). The number of differentially expressed genes (DEGs) showed first an increase and then a decrease during fruit development (Fig. 4b). The number of DEGs was most abundant in the middle and later stages of development in X versus F (150–170 DAFB).

KEGG pathway analysis was used to ascertain the potential involvement of metabolic pathways in the regulation of fruit aroma volatile synthesis. Since the majority of volatile aroma compounds were synthesized, and the numbers of DEGs most abundant, in the middle and later stages of fruit development, we selected comparisons of X3 versus F3 and X3 versus P3 (‘Ruixue’, ‘Fuji’ and ‘Pink Lady’ fruit at 170 DAFB) for KEGG pathway analysis (Supplementary Table 1; Fig. 4b). The DEGs mapped to 110 KEGG pathways in the pairwise comparison of X3 versus F3, with the greatest number of DEGs mapped to phenylpropanoid biosynthesis (mdm00940) (Fig. 4c). Volatile aroma compound synthesis pathways included sesquiterpenoid and triterpenoid biosynthesis, and fatty acid degradation was also significantly enriched in KEGG. A comparison (X3 versus P3)of DEGs resulted in the identification of 114 KEGG pathways, with significant enriched pathways putatively identified as plant-pathogen interaction (mdm04626), phenylpropanoid biosynthesis (mdm00940) and sesquiterpenoid and triterpenoid biosynthesis (mdm00909) (Fig. 4d). In general, the KEGG enrichment analyses indicated that fatty acid metabolism, phenylpropanoid metabolism and sesquiterpenoid metabolism play a critical role in fruit aroma volatile synthesis in ‘Ruixue’, ‘Fuji’ and ‘Pink Lady’.

### Expression changes in volatile compounds -related genes involved in fatty acid, isoleucine and sesquiterpenoid metabolism pathways

**Fig. 5:**
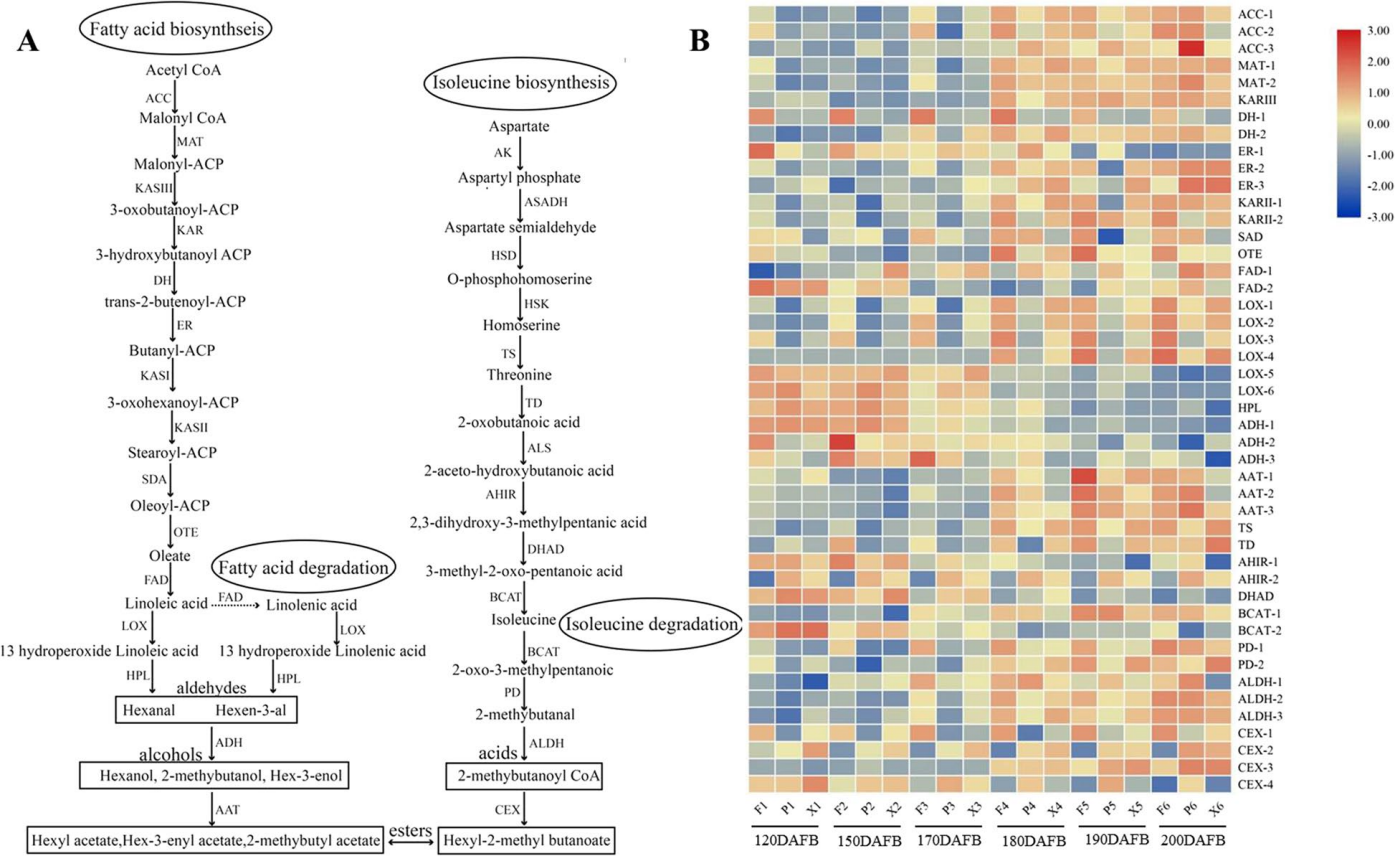
Expression profiles of volatile -related genes for ester biosynthesis associated with fatty acid and isoleucine pathway during fruit development. **a** Biosynthetic pathway of ester involve in fatty acid and isoleucine pathway. **b** Expression profiles of volatile -related genes for ester biosynthesis. ACC: Acetyl-CoA carboxylase; MAT: Malonyl-CoA ACP transacylase; KASIII: 3-Ketoacyl ACP synthase III; KAR: 3-Ketoacyl ACP reductase; DH: 3-Hydroxyacyl ACP dehydratase; ER: 2,3-Trans-enoyl ACP reductase; KASI: 3-Ketoacyl ACP synthase I; KASII: 3-Ketoacyl ACP synthase II; SAD: Stereate ACP desaturase; OTE: Oleate-ACP thioesterase; FAD: Oleate and linoleate desaturase; AK: Aspartate kinase; ASADH: Aspartate semialdehyde dehydrogenase; HSD: Homoserine dehydrogenase; HSK: Homoserine kinase; TS: Threonine synthase; TD: Threonine deaminase; ALS: Acetolactic synthetase; AHIR: Acetohydroacid isomeroreductase; DHAD: Dihydroxy acid dehydratase; BCAT: Branched chain aminotransferase; LOX: Lipoxygenase; HPL: Hydroperoxide lyase; PD: Pyruvate decarboxylase; ADH: Alcohol dehydrogenase; ALDH: Aldehyde dehydrogenase; AAT: Alcohol acyl transferase; CXE: Carboxylesterase

Esters (straight and branched chain esters) are among the most abundant volatile compounds in apple fruits. Straight chain esters are produced by the fatty acid metabolism pathway while branched chain esters are produced by the isoleucine metabolism pathway (Fig. 5a and Supplementary Table S3). The expression levels of numerous genes showed significant increases, but were differently expressed in the different cultivars during fruit development (Fig. 5b). DH-1(3-hydroxyacyl ACP dehydratase) exhibited a very low expression level in ‘Ruixue’, significantly lower than in ‘Fuji’ during fruit development. Interestingly, ER-1 and ER-2 (2, 3-trans-enoyl ACP reductase) had an opposite expression pattern. Lipoxygenases (LOX) are involved in the initial steps of ester biosynthesis in fatty acid degradation, including six genes (LOX-1 to LOX-6). Four lipoxygenases (LOX-1 to LOX-4) dramatically increased during fruit development, transcribed at high levels in ‘Ruixue’ but at low levels in ‘Pink Lady’. However, LOX-5 and LOX-6 showed high expression levels prior to 170 DAFB then decreased rapidly. Hydroperoxidelyase (HPL) is the final step toward biosynthesis of aldehydes (hexanal) with an expression level that first increased then decreased. Alcohol dehydrogenase reduced aldehydes from the fatty acid and isoleucine degradation pathways to alcohols. Transcript abundance of ADH-1, ADH-2 and ADH-3 continuously decreased during fruit development. Alcohol acyl transferases (AAT) are the rate limiting enzymes for ester biosynthesis. Transcript abundance of AAT-1, AAT-2 and AAT-3 continuously increased during fruit development. It is noteworthy that the transcript abundance of AAT in ‘Ruixue’ was lower than in ‘Fuji’ and ‘Pink Lady’. Aldehyde dehydrogenase (ALDH-1 to ALDH-3) and carboxylesterase (CXE-1 to CXE-2) are important enzymes in biosynthesis of branched chain esters by the isoleucine metabolism pathway and were differentially expressed during fruit development.

**Fig. 6:**
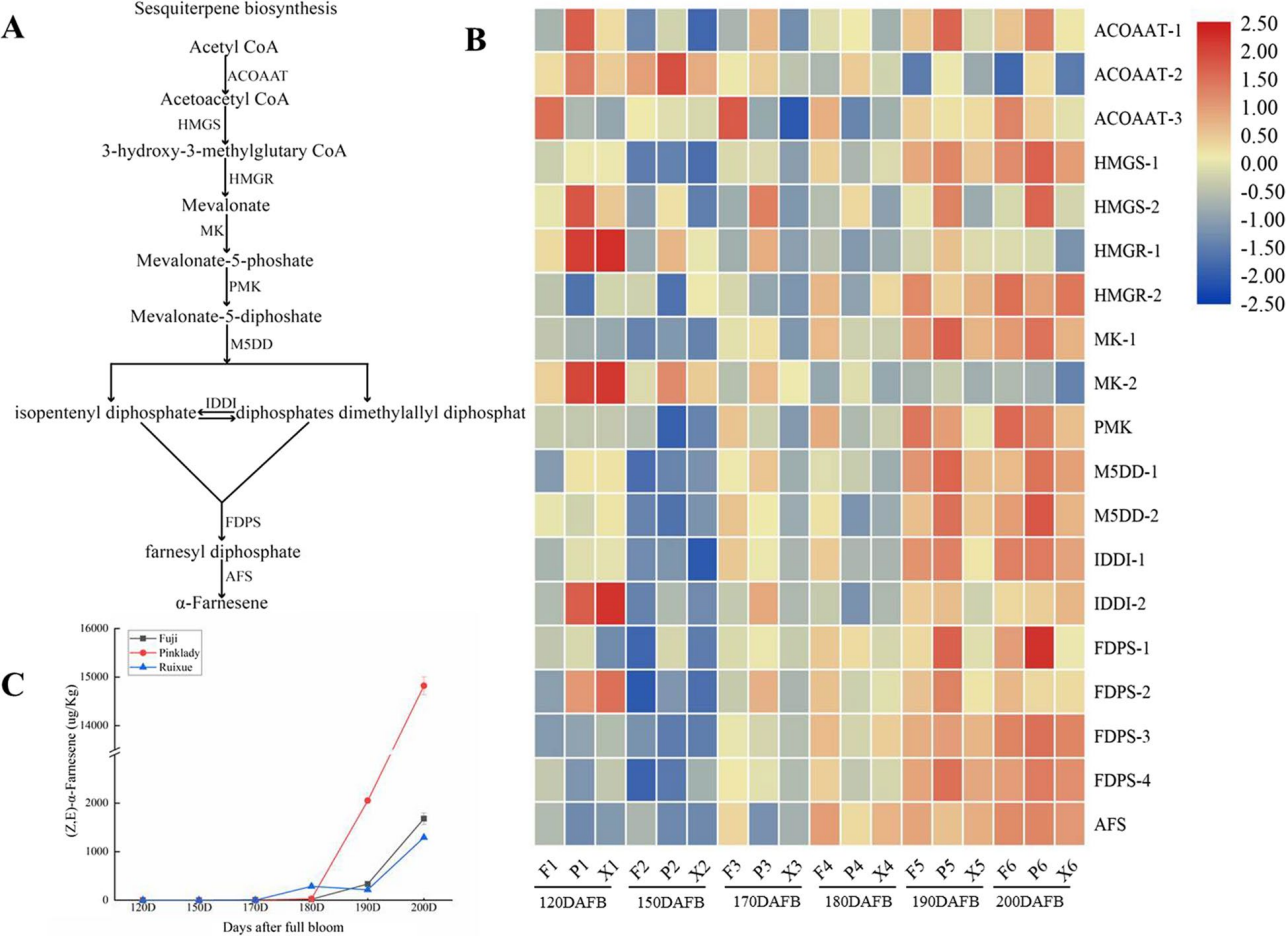
Expression profiles of volatile-related genes for sesquiterpenes biosynthetic pathway during fruit development. **a** Biosynthetic pathway of sesquiterpenes. **b** Expression profiles of volatile-related genes for sesquiterpenes biosynthesis. ACOAAT: Acetyl-CoA acetyltransferase; HMGS: HMG-CoA synthase; HMGR: HMG-CoA reductase; MK: Melalonate kinase; PMK: Phosphomevalonate kinase; M5DD: Mevalonate 5-diphosphate decarboxylase; IDDI: Isopentyl-diphosphate Δ-isomerase; PPS: Polyprenyl synthase; AFS: α-Farnesene synthase. **c** The change in α-Farnesene of Ruixue’ and its parents apples during fruit development

Sesquiterpenes are the most prominent terpenoids in apples. The main sesquiterpene that accumulated was (Z, E)-α-farnesene, the most abundant of any aroma volatile compounds (Supplementary Table S1). Sesquiterpenes are produced by the mevalonate (MVA) pathway in the cytoplasm in nine enzymatic steps (Fig. 6a). Expression levels of numerous genes showed significant increases during fruit development. There were different expressions of sesquiterpene biosynthetic genes in the different cultivars during fruit development. Notably, the expression levels of α-farnesene synthase (AFS) exhibited drastic increases, higher in ‘Pink Lady’ than in ‘Fuji’ and ‘Ruixue’ (Fig. 6b). Similarly, α-Farnesene exhibited rapid increases during fruit development with the same expression pattern as AFS (Fig. 6c).

### Expression changes in potential transcriptional factors regulating volatile synthesis

Transcriptional factors play a crucial role in regulating aroma volatile synthesis. To clarify potential transcription factors that may be involved in regulating volatile biosynthesis, we further analysed expression patterns of transcription factors during fruit development, including MYC2, ERF, WRKY, MYB, BZIP and MADS-box TFs. The expression patterns of numerous transcription factors increased during fruit development, but were differently expressed in the different cultivars (Supplementary Fig. S1 and Table S3).

### Quantitative reverse transcriptase-PCR validation of the transcriptome data

**Fig. 7:**
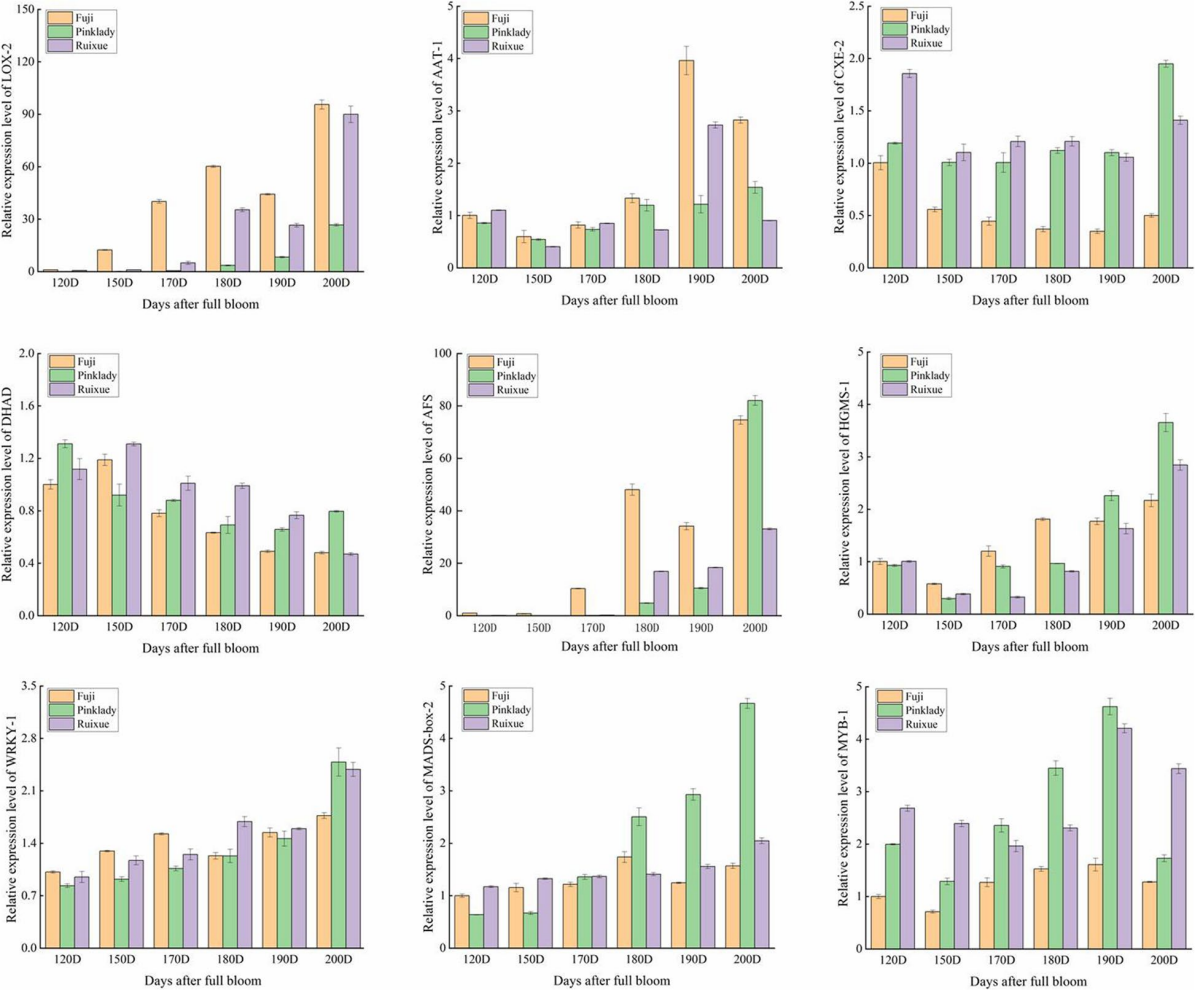
qRT-PCR validated candidate genes in Ruixue’ and its parents apples during fruit development. Error bars show ± SE from three biological replicates

To validate the reliability and repeatability of the transcriptome data, 6 aroma biosynthesis and 3 transcription factor genes were selected for analysis of their expression levels using qRT-PCR. Gene-specific primers used in this analysis are listed in Supplementary Table S4. The expression profiles of 9 candidate genes generated using qPCR were very similar to the RNA-Seq results (RPKM values), which had high a Pearson correlation coefficient (*R*^2^ > 0.9). These results indicate that transcriptomic data were accurate and reproducible (Fig. 7).

### Correlation analysis for volatiles, biosynthesis gene and transcriptional factors in ‘Ruixue’ during fruit development

**Fig. 8:**
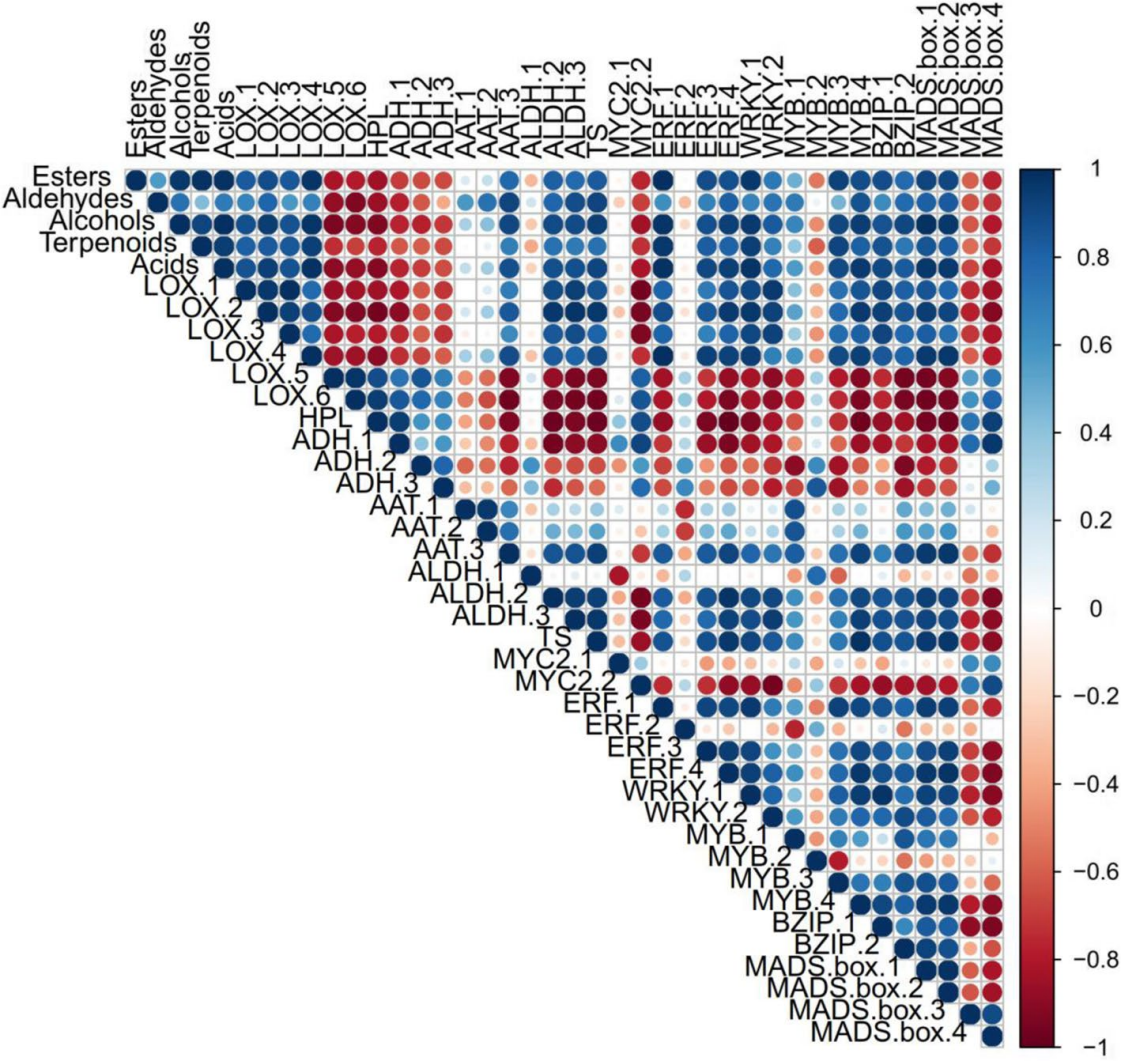
Correlation analysis for volatile compounds, biosynthesis gene involved in the lipoxygenase (LOX), mevalonate (MVA) pathway and candidate transcription factors in ‘Ruixue’ during fruit development

To understand the Correlation network between the transcriptome and volatilome, correlation analysis between volatile compounds biosynthesis gene involved in the lipoxygenase (LOX), mevalonate (MVA) pathway and candidate transcription factors was carried out (Fig. 8). For this analysis, the mean content of the volatile compounds groups (esters, aldehydes, alcohols, terpenoids and acid) and the mean expression levels of expressed transcripts were used for the correlation tests (Spearman rank correlation test). Among the biosynthesis genes, expression pattern of LOX-1, LOX-2, LOX-3 and LOX-4 had a positive correlation with total content of esters, aldehydes and alcohols, terpenoids and acid, particularly LOX1 with the highest correlation coefficient. However, expression pattern of HPL, ADH-, ADH-2 and ADH-3 had a negative correlation with total content of esters, aldehydes and alcohols, terpenoids and acid. Among the candidate transcription factors, the gene with the highest correlation coefficient (*r* > 0.99) between its expression pattern and content of esters alcohols, terpenoids and acid was ERF-1, which is an ethylene response factor, followed by WRKY-1. Taken together, these results indicate that partial participation of the LOX pathway and mevalonate (MVA) in the metabolism of volatile compounds in ‘Ruixue’ during fruit development.

## Discussion

### Dynamic variations of volatile compounds profiles of ‘Ruixue’ and its parents apples during fruit development

Volatile compounds are a crucial indicator of fruit flavour that has been used to distinguish varieties of fruit and fruit cultivars [3]. Profiles of apple volatile compounds are extremely complex, with aroma composition and concentration influenced by cultivar-specific traits and fruit development stages [5]. The profiles of volatile compounds change with ripening. Aldehydes are dominant volatile compounds in the early stages of fruit development [19], but their content decreases with fruit ripening followed by dramatic increases in alcohols, and finally esters are dominant at fruit maturation [2].

The volatile compounds of ‘Ruixue’, ‘Pink Lady’ and ‘Fuji’ apples were determined during fruit development. A total of 40, 46 and 32 volatile compounds were putatively detected in ‘Ruixue’, ‘Pink Lady’ and ‘Fuji’ apples, respectively, including esters, aldehydes, alcohols, terpenoids and acids. Aldehydes were the predominant volatiles and account for more than 90% of total volatiles in the three cultivars during the early stages of fruit development. Alcohols and esters showed a significant increase during the latter stages of fruit development in all cultivars. A total of 54 volatiles putatively detected in ‘Ruixue’, ‘Fuji’ and ‘Pink Lady’ during fruit development clustered into seven groups (A, B, C, D, E, F and G) in the Heatmap dendrogram. Group A, consisting of two aldehydes AL1 (Hexanal) and AL3 (2-Hexenal), was a difference in content of AL1 and AL3 of ‘Ruixue’ compare to its parents. In group B, including six compounds, among them, E26 (Acetic acid, butyl ester) and E28 (Acetic acid, pentyl ester) were the most significant difference in three cultivars, which were more abundant in its parents, while only a few could be detected in ‘Ruixue’ fruit. In Group C, including 8 esters and 1aldehyde (2-Heptenal, (Z)-), all of esters were lower in ‘Ruixue’ fruit than that in its parents. In group E and F, the majority of ester volatiles were abundant in ‘Ruixue’ and ‘Fuji’ apple fruit. Altogether, the ester and aldehydes of the ‘Ruixue’ cultivar and its parents were dominant volatiles groups. Our data show that esters and terpenoids were the main volatiles in ripening fruit of ‘Pink Lady’ and ‘Fuji’ apples, mainly including butyl 2-methylbutanoate; propanoic acid, hexyl ester; propanoic acid, hexyl ester; hexanoic acid, hexyl ester; acetic acid, hexyl ester and (Z, E)-α-farnesene. Among these, acetic acid, hexyl ester was putatively identified as a characteristic volatile compound [20]; however, acetic acid, hexyl ester was not putatively detected in ‘Ruixue’ apples. Interestingly, aldehydes were the dominant volatile compounds in ripening fruit of ‘Ruixue’, mainly including 2-hexenal; 2-hexenal; octanal; (E)-2-octenal and nonanal. In addition, 1-butanol, 2-methyl- in ‘Ruixue’ was significantly higher than in ‘Pink Lady’ and ‘Fuji’ apples, contributing to a sweetish sensation [17]. Altogether, these results show that aldehyde-type flavours predominate in ‘Ruixue’, while ‘Pink Lady’ and ‘Fuji’ exhibit ester-type flavours. It has been shown that esters are the most important contributors to fruit flavour, accounting for more than 80% of the total volatile compounds in ‘Granny Smith’ [21], ‘Gala’ and ‘Starking Delicious’ apples [22, 23]. Similar studies show that esters are important volatile compounds that significantly affect fruit flavour in bananas and strawberries [24–26]. However, our data show that the aldehydes and terpenoids are major volatiles groups in ‘Ruixue’ fruit, further studies will be required to clarify the roles of these aldehydes and terpenoids volatile compounds that affect fruit flavour using chromatography–olfactometry (GC-O) and Sensory Evaluation [4]. Sweetness of fruit is one of the primary drivers of consumer preference. It has been shown apple sweetness is provided by some volatile compounds, mainly esters and farnesene [27]. Generally, ‘Ruixue’ apple fruit in fruit firmness, total soluble solids and titrable acidity have differences from its parents apples during fruit development. Taken together, the difference of the sugar, acid and volatile in ‘Ruixue’ apple and its parents together may determine diverse flavour of the fruit.

### Analysis of expression patterns of volatile compounds—related genes during fruit development involved in fatty acid, isoleucine and sesquiterpenoid metabolism pathways

Previous studies have shown that numbers of volatile compounds are synthesized mainly by the fatty acid, isoleucine, mevalonate and phenylpropanoid metabolism pathways [5]. Among these, fatty acid and isoleucine metabolism are the most important pathways for synthesis of aldehydes, alcohols and esters, associated with β-oxidation and lipoxygenase activity (LOX). The lipoxygenase pathway mainly comprises four crucial enzymes including lipoxygenase (LOX), hydroperoxidelyase (HPL), alcohol dehydrogenase (ADH) and alcohol acyl transferase (AAT) [6]. The large number of differential expression of these genes (DEGs) in the fatty acid, isoleucine and sesquiterpenoid metabolism pathways may affect the accumulation of volatile aroma compounds. It has been shown that lipoxygenases (LOX) are involved in the initial steps toward biosynthesis of aldehydes, alcohols and esters involved in fatty acid degradation, with linoleic and linolenic being two important substrates for LOX [28]. Twenty-three, eleven putative LOX genes were putatively identified in Golden Delicious and Royal Gala apples, respectively [5, 7]. Recent studies show that *MdLOX1a* and *MdLOX5e* are involved in fruit volatile production, arguing for a molecular breeding approach [7]. In our study, six differentially expressed genes (LOX-1 to LOX-6) were putatively identified in ‘Ruixue’, ‘Fuji’ and ‘Pink Lady’. Among these, four lipoxygenases (LOX-1 to LOX-4) dramatically increased during fruit development, transcribed at high levels in ‘Ruixue’ but at low levels in ‘Pink Lady’. These observations are in agreement with increases in aldehydes during fruit development and aldehyde concentrations in ‘Ruixue’ fruit greater than those in ‘Pink Lady’, suggesting that LOX-1, LOX-2(*MdLOX1a*), LOX-3 and LOX-4 play a key positive role in the biosynthesis of aldehydes. Similar results were reported previously in Korla fragrant pears (Pyrus sinkiangensis Yu) [29]. However, LOX-5 and LOX-6 first increased and then rapidly decreased. Interestingly, the expression of LOX1 was higher than LOX-2(*MdLOX1a*), which is involved in fruit volatile production [7]. Further functional studies will be required to clarify the regulatory roles of LOX1 in regulating apple volatile biosynthesis. The differences of LOX expression level in ‘Ruixue’ fruit and its parents may cause diverse of aldehydes, which was consistent with results of volatiles profiles in Fig. 2. Alcohol dehydrogenase (ADH) reduced aldehydes from the fatty acid and isoleucine degradation pathways to alcohols [2]. It has been shown that ADH activity decreased in ‘Fuji’ apples during fruit development [21]. Conversely, ADH activity increased until harvest in ‘Jonagold’ apple [30]. In addition, transcript levels of ADH decreased during fruit development [29]. Here we have identified three ADH genes and our results are similar to these previous studies in that transcript abundances of ADH-1, ADH-2 and ADH-3 continuously decreased during fruit development. However, content of the alcohols compounds showed an increasing trend during fruit development. These results indicate that regulation of ADH occurs at the post-transcriptional and translational level [8]. Alcohol acyl transferases (AAT) are the rate limiting enzymes of ester biosynthesis. Studies have shown that AAT activity increases with fruit ripening in ‘Granny Smith’ and ‘Fuji’ apples [13]. Transcript levels of *AAT1-GS* and *AAT1-RG* increased during fruit development in ‘Granny Smith’ and ‘Royal Gala’ apples [31]. Our data also show that transcript abundances of AAT-1, AAT-2 and AAT-3 continuously increase during fruit development. It is also notable that the transcript abundance of AAT in ‘Ruixue’ was lower than in ‘Fuji’ and ‘Pink Lady’. This is consistent with the observation that the ester content of ‘Ruixue’ is lower than in ‘Fuji’ and ‘Pink Lady’. Terpenes are produced from the mevalonate (MVA) pathway in the cytoplasm [5]. Terpene synthases (TPS) are the key enzymes for synthesising terpene volatiles. To date, 55 putative apple TPSs have been putatively identified and only 10 TPS genes are predicted to be functional, able to synthesize most of the terpene volatiles produced in the ‘Royal Gala’ such as D-germacrene, linalool and a-pinene. However, we putatively detected only the terpene volatile α-farnesene (sesquiterpenes), synthesized by α-farnesene synthase (MdAFS1). Expression levels of α-farnesene synthase (AFS) drastically increase during fruit development and were higher in ‘Pink Lady’ than in ‘Fuji’ and ‘Ruixue’. Differentially expressed aroma-related genes may be responsible for changes in ‘Ruixue’ and its parents apples volatile compounds. Altogether, these results show that aroma synthesis is a complex and precise process that involves multiple structural gene families and metabolic pathways.

### Expression changes in candidate transcription factors that may regulate volatile biosynthesis

Previous studies have shown that transcription factors (TFs) play a primary role in regulating volatile plant secondary metabolites [32]. To clarify potential transcription factors that may be involved in regulating volatile biosynthesis, we further analysed expression patterns of transcription factors during fruit development, including MYC2, ERF, WRKY, MYB, BZIP and MADS-box TFs. It has been reported that *AtMYC2*, a bHLH transcription factor, promotes sesquiterpene production by binding to promoters of *TPS21* and *TPS11* in *Arabidopsis thaliana* flowers then activates their transcription, mediating both GA and JA signals [32]. Here, we have identified two MYC2 genes (MYC2-1 and MYC2-2). Transcript levels of MYC2-1 were high during early and late stages of fruit development. This indicates that MYC2-1 may be involved in regulating aldehyde synthesis during the early stages and ester synthesis during the late stages of fruit development. However, transcript abundances of MYC2-2 maintained a lower level during fruit development. Research has shown that ERF1 and ERF2 of AP2/ERF transcription factors are involved in regulating sesquiterpene synthase amorpha-4,11-diene synthase [33]. In oranges, *CitAP2.10* promotes ( +)-valencene content via activation of the terpene synthase CsTPS1 [14]. Recently, an ethylene response factor (FaERF#9) has been shown to activate FaQR transcripts and to up-regulate furanone biosynthesis in strawberries [34]. These results indicate that ERF predominantly regulates terpene biosynthesis. Our results show that transcript abundances of ERF-1, ERF-1, ERF-3 and ERF-4 increased during fruit development; however, the transcript abundance of ERF-2 first increased then decreased. In accordance with previous studies and our data, ERF may be involved in regulating (Z, E)-α-farnesene biosynthesis in apples. It has been reported that *GaWRKY1*, WRKY transcription factors regulate sesquiterpene biosynthesis in cotton via activation of the ( +)—δ-cadinene synthase (CAD1) transcript [35]. In addition, the MYB transcription factor (FaMYB98) up-regulates furanone biosynthesis in strawberries [34]. Therefore, further functional studies will be required to clarify the regulatory roles of these transcription factors in apple volatile biosynthesis.

## Conclusions

The volatiles profile of ‘Ruixue’ and its parents apples during fruit development were putatively identified using SPME–GC–MS. Our results show that the profile of volatile compounds changes with ripening. Aldehydes were dominant volatile compounds during the early stages of fruit development. In ripe fruit, esters and terpenoids were the main aroma volatiles in ripening fruit of ‘Pink Lady’ and ‘Fuji’ apples, and they included butyl 2-methylbutanoate; propanoic acid, hexyl ester; propanoic acid, hexyl ester; hexanoic acid, hexyl ester; acetic acid, hexyl ester and (Z, E)-α-farnesene. Interestingly, aldehydes and terpenoids were the dominant volatile aroma compounds in ripening fruit of ‘Ruixue’, and they mainly included hexanal; 2-hexenal; octanal, (E)-2-octenal; nonanal and (Z, E)-α-farnesene. By comparing the transcriptome profiles of ‘Ruixue’ and its parents during development, we identified a large number of aroma-related genes involved in the fatty acid, isoleucine and sesquiterpenoid metabolism pathways and identified transcription factors that may regulate aroma biosynthesis. Our initial study facilitates a better understanding of the volatile aroma compounds that affect fruit flavour as well as the mechanisms underlying differences in flavour between ‘Ruixue’ and its parents.

## Methods

### Plant material

Fruit of three apple cultivars, new cultivar ‘Ruixue’ (An application will be made to protect ‘Ruixue’ nationally in China. Trees and bud wood under rules of international Plant Variety Rights are available for research purposes, which can be obtained from Zhengyang Zhao at the College of Horticulture, Northwest A&F University, Yangling, Shaanxi, China. The number of cultivar: CNA20151469.1), ‘Pink Lady’ and ‘Fuji’, were collected from the national apple model of Northwest A&F University in Baishui County, Weinan City, Shaanxi Province, China. The three apple cultivars (6-year-old) were grafted onto M26 rootstock and planted in 4.0 × 1.5 m plots. Fruit of uniform appearance, without mechanical damage and disease were collected from ‘Ruixue’, ‘Pink Lady’ and ‘Fuji’ at six different developmental stages, 120, 150, 170, 180, 190 and 200 days after full bloom (DAFB). Three independent replicates were performed for each sample, consisting of 10 fruits each from 15 apple trees. A total of 54 fruit samples (three cultivars × six developmental stages × three biological replicates) were frozen in liquid nitrogen then stored at -80˚C for GC–MS and RNA-Seq analysis.

### Mensuration of fruit physiological characteristics

Firmness of flesh was measured using a GS-15 fruit texture analyser (Guss Manufacturing Pty. Ltd., Cape Town, South Africa) on both sides of the fruit equator of peeled fruits (test depth, 8 mm; diameter probe,10 mm). Total soluble solids (TSS) were measured using a hand-held refractometer (Pocket PAL-1, ATAGO, Tokyo, Japan). Titrable acidity (TA) was measured using a digital fruit acidity meter (GMK-835F Perfect, Germany). All samples were measured for each cultivar (‘Ruixue’, ‘Pink Lady’ and ‘Fuji’) at each time point (120, 150, 170, 180, 190 and 200 DAFB).

### Analysis of volatile aroma compounds using gas chromatography-mass spectrometry (GC–MS)

Solid phase micro extraction (SPME) was used to extract the volatile compounds of fruit with three biological replicates for each sample [36]. Approximately 5.0 g frozen fruit (powder), 1.0 g NaCl and 10 μL 3-nonanone(0.04 μL mL^−1^)as an internal standard sealed in a 50 mL vial, then the headspace gases were extracted for 40 min at 40 °C using fibre coated with 50/30 μm divinylbenzene/carboxen/polydimethylsiloxane (DVB/CAR/PDMS). Subsequently, samples were directly injected into the injection port of a GC–MS (Thermo Fisher Scientific Inc., USA, ISQ, TRACE GC ULTRA) with a splitless mode for 2.5 min at 230 °C. Volatile analysis was performed using a HP-INNO wax column (60 m × 0.25 mm inner diameter × 0.25 μm film thickness) with a carrier gas helium flow rate of 34 cm s^−1^. The initial oven temperature was maintained at 40 °C for 3 min, followed by a temperature increase at a rate of 5 °C min^−1^ to 150 °C, then to 220 °C at a rate of 10 °C min^−1^ then maintained at 220 °C for 5 min. The energy of electron impact (EI) was 70 eV. The transfer—temperature and source—temperature were 240 °C. Aroma volatiles were qualitatively analysed by comparison with mass spectra (MS) from NIST2014 Library. Aroma volatiles were relatively quantified using the peak area of the internal standard (3-nonanone).

### RNA extraction and RNA sequencing analysis

A total of 54 samples (three cultivars × six developmental stages × three biological replicates) were subjected to transcriptome analyses. Total RNA was extracted from fruit of ‘Ruixue’, ‘Pink Lady’ and ‘Fuji’ at 120, 150, 170, 180, 190 and 200 DAFB using the CTAB method [37]. mRNA (containing polA) was isolated from total RNA using magnetic beads with oligo (dT). Fifty-four cDNA libraries were constructed using the NEBNext® UltraTM RNA Library Prep Kit for Illumina® (NEB, USA) and sequenced on the Illumina HiSeq platform. Low-quality reads and N-containing reads were removed from the raw RNA-seq reads.

Transcript abundance was quantified using HISAT2 software and normalized using the fragments-per-kilobase-of-transcript-per-million-mapped-reads (FPKM) method. Differentially expressed genes (DEGs) were putatively identified using DESeq2 with log2 (fold change) ≥ 1 and corrected P ≤ 0.005. All identified DEGs were subjected to gene-ontology (GO) enrichment and KEGG pathway analysis with padj ≤ 0.05 [38, 39].

### Quantitative reverse transcriptase-PCR validation of the transcriptome data

Total RNA was extracted using the CTAB method [23]. qPCR was performed using a StepOneplus™ instrument (Thermo Fisher Scientific Inc., USA). Real-time PCR reaction volumes of 10 μL included 5 μL 2 × SYBR Green PCR Master Mix (Vazyme), 1 μL of cDNA, 0.4 μL of each primer, 0.2 μL 50 × ROX Reference and 3.4 μL distilled deionized H_2_O (ddH_2_O). Gene-specific primers (Primer Premier 6.0) used in the qRT-PCR analysis are listed in Supplementary Table S4. The qPCR protocol: 95 °C for 5 min; 40 cycles of 95 °C for 15 s and 60 °C for 30 s. *MdEF-1α* was used as a reference gene to normalize all gene expression levels. The 2^−ΔΔCt^ method was used to calculate relative expression levels of genes [40].

### Statistical analysis

Figures were drawn using ORIGIN 2019 (Microcal Software, Inc., Northampton, MA). Significant difference analyses (*P* < 0.05) were carried out using one-way ANOVA with Duncan’s test (SPSS 19.0, SPSS Inc., Chicago, IL). Gene ontology (GO) enrichment of the differential expressed genes (DEGs), Kyoto Encyclopedia of Genes and Genomes (KEGG) enrichment analysis of the DEGs, PCA (principal component analysis) and Correlation analysis were performed using Tbtools Version 1.075 software [41–43].

## Supplementary Information

**Additional file 1: Supplemental Table S1.** Changes in volatiles content of‘Ruixue’ and its parents apples during fruit development. “RX” represents ‘Ruixue’; “FJ” represents ‘Fuji’; “PL” represents ‘Pink Lady’. The six different fruit developmental stages of‘Ruixue’, ‘Pink Lady’ and ‘Fuji’, namely at 120, 150, 170, 180, 190 and 200 DAFB (days after full bloom ). “–” represents no detected. Means with different letters are significantly different at P <0.05, Duncan’s new multiple range test. **Supplemental Table S2.** Throughput and quality of RNA-seq in ‘Ruixue’ and its parents apples after filter. **Supplemental Table S3.** Analyzed expression level of volatile-related genes of the FPKM values in Ruixue’ and its parents apples during fruit development. “F1, F2 F3, F4, F5 and F6” respectively represents at 120, 150, 170, 180, 190 and 200 DAFB(days after full bloom ) of ‘Fuji’; “P1, P2, P3, P4, P5 and P6 ” respectively represents1 at 120, 150, 170, 180, 190 and 200 DAFB(days after full bloom ) of ‘Pink Lady’; “X1, X2, X3, X4, X5 and X6” respectively represents at 120 ,150,170,180,190 and 200 DAFB(days after full bloom ) of ‘Ruixue’. Means with different letters are significantly different at *P* <0.05, Duncan’s new multiple range test. **Supplemental Table S4.** Gene-specific primers used for RT-qPCR analysis. **Supplemental Fig. S1.** Analyzed expression patterns of transcription factors in Ruixue’ and its parents apples during fruit development by Heatmap.

## Data Availability

The datasets generated and/or analysed during the current study are available in the NCBI database repository, the accession number is: PRJNA728501. All data generated or analyzed during this study are included in this published article and its supplementary information files.

## Abbreviations

mRNA: Messenger RNA
MYC2: Myelocytomatosis protein 2
bHLH: Basic Helix-Loop-Helix
qRT-PCR: Quantitative reverse transcriptase polymerase chain reaction
CTAB: Cetyltrimethylammonium bromide
RNA: Ribonucleic acid
cDNA: Complementary deoxyribonucleic acid
