# Supplementary material for: Transcriptome and metabolite profiling analyses provide insight into volatile compounds of the apple cultivar ‘Ruixue’ and its parents during fruit development
Source: BMC Plant Biol. 2021 May 24;21:231. doi: 10.1186/s12870-021-03032-3 (PMC8147058; doi:10.1186/s12870-021-03032-3)

**Appendix A. Supplementary data**

**Supplemental Table S1** Changes in volatiles content of‘Ruixue’ and its parents apples during fruit development. “RX” represents ‘Ruixue’; “FJ” represents ‘Fuji’; “PL” represents ‘Pink Lady’. The six different fruit developmental stages of‘Ruixue’, ‘Pink Lady’ and ‘Fuji’, namely at 120, 150, 170, 180, 190 and 200 DAFB (days after full bloom ). “–” represents no detected. Means with different letters are signiﬁcantly different at P <0.05, Duncan’s new multiple range test.

**Supplemental Table S2** Throughput and quality of RNA-seq in ‘Ruixue’ and its parents apples after filter.

**Supplemental Table S3** Analyzed expression level of volatile-related genes of the FPKM values in Ruixue’ and its parents apples during fruit development. “F1, F2 F3, F4, F5 and F6” respectively represents at 120, 150, 170, 180, 190 and 200 DAFB(days after full bloom ) of ‘Fuji’; “P1, P2, P3, P4, P5 and P6 ” respectively represents1 at 120, 150, 170, 180, 190 and 200 DAFB(days after full bloom ) of ‘Pink Lady’; “X1, X2, X3, X4, X5 and X6” respectively represents at 120 ,150,170,180,190 and 200 DAFB(days after full bloom ) of ‘Ruixue’. Means with different letters are signiﬁcantly different at P <0.05, Duncan’s new multiple range test.

**Supplemental Table S4** Gene-speciﬁc primers used for RT-qPCR analysis.

**Supplemental Fig. S5** Analyzed expression patterns of transcription factors in Ruixue’ and its parents apples during fruit development by Heatmap.

**Table S1**

| **RT** | **RI** | **Stage** | | 120DAFB | | | 150DAFB | | | 170DAFB | | | 180DAFB | | | 190DAFB | | | 200DAFB | | |
| --- | --- | --- | --- | --- | --- | --- | --- | --- | --- | --- | --- | --- | --- | --- | --- | --- | --- | --- | --- | --- | --- |
|  |  | **volatile**（ug/Kg） | | RX | FJ | PL | RX | FJ | PL | RX | FJ | PL | RX | FJ | PL | RX | FJ | PL | RX | FJ | PL |
|  |  | **Esters** | **Code** |  |  |  |  |  |  |  |  |  |  |  |  |  |  |  |  |  |  |
| 17.27 | 1285 | 2-Methylbutyl 2-methylbutyrate | E1 | - | - | - | - | - | - | - | 9.1^c^ | - | 11.34^c^ | 63.51^a^ | - | 9.46^c^ | 27.8^b^ | - | 27.5^b^ | 28.15^b^ | - |
| 12.7 | 1014 | Propanoic acid, 2-methyl-, pentyl ester | E2 | - | - | - | - | - | - | - | - | - | 13.7^b^ | - | - | 18.12^a^ | - | - | - | - | - |
| 13.35 | 1105 | Butanoic acid, 2-methyl-, propyl ester | E3 | - | - | - | - | - | - | - | 37.29^b^ | - | - | 49.79^a^ | - | 15.23^c^ | 16.36^c^ | - | 11.77^d^ | 45.05^a^ | - |
| 16.06 | 1196 | Butyl 2-methylbutanoate | E4 | - | - | - | - | - | - | - | 25.52^e^ | - | 31.32^d^ | 228.5^a^ | - | 27.11^e^ | 132.29^b^ | 75.34^c^ | 51.36^c^ | 158.33^b^ | 245.49^a^ |
| 18.65 | 1363 | Butanoic acid, 2-methyl-, pentyl ester | E5 | - | - | - | - | - | - | - | - | - | 7.7^e^ | 28.13^b^ | - | 7.79^e^ | 17.81^d^ | - | 16.48^d^ | 20.39^c^ | 37.95^a^ |
| 22.33 | 2034 | Hexanoic acid, 2-methylbutyl ester | E6 | - | - | - | - | - | - | - | - | - | - | 36.37^b^ | - | - | 20.14^c^ | 25.13^c^ | 32.64^b^ | 20.09c | 51.45^a^ |
| 12.86 | 1123 | 1-Butanol, 2-methyl-, acetate | E7 | - | 15.92^g^ | - | - | 112.36^g^ | 15.22^g^ | - | 438.04^d^ | - | - | 1655.62^a^ | 57.5^f^ | - | 835.12^a^ | 353.88^e^ | - | 864.45^b^ | 545.37^c^ |
| 13.41 | 1153 | Propanoic acid, butyl ester | E8 | - | - | - | - | - | - | - | - | - | - | 124.14^a^ | - | 14.19^f^ | 71.84^c^ | 47.59^d^ | 26.04^e^ | 107.42^b^ | 86.27^c^ |
| 18.99 | 1344 | Propanoic acid, hexyl ester | E9 | - | - | - | - | - | - | - | 17.86^d^ | - | 5.37^e^ | 93.31^b^ | - | 7.44^e^ | 47.22^c^ | 102.97^b^ | 39.79^c^ | 98.68^b^ | 282.29^a^ |
| 10.66 | 1045 | Propanoic acid, propyl ester | E10 | - | - | - | - | - | - | - | - | - | - | - | - | - | - | - | - | 24.21 | - |
| 14.68 | 1241 | Propanoic acid, pentyl ester | E11 | - | - | - | - | - | - | - | 34.11^b^ | - | - | 42.46^a^ | - | 5.31^e^ | 22.77^c^ | - | 18^d^ | 24.22^c^ | - |
| 12.9 | 1156 | Butanoic acid, propyl ester | E12 | - | - | - | - | - | - | - | - | - | - | - | - | - | - | - | 14.59 | - | - |
| 15.69 | 1220 | Butanoic acid, butyl ester | E13 | - | - | - | - | - | - | - | 12.01^e^ | - | 12.62^e^ | 82.37^c^ | 19.31^f^ | 19.19^f^ | 59.54^d^ | 71.16^d^ | 43.64^e^ | 104.14^b^ | 158.23^a^ |
| 12.21 | 1192 | Butanoic acid, hexyl ester | E14 | - | - | - | - | - | - | - | - | - | 20.39^g^ | 90.33^d^ | 36.46^f^ | 26.86^g^ | 57.85^e^ | 167.83^b^ | 136.11^c^ | 127.63^c^ | 544.06^a^ |
| 10.43 | 1043 | Butanoic acid, ethyl ester | E15 | - | - | - | - | - | - | - | - | - | - | - | - | - | - | - | - | 15.98 | - |
| 17.05 | 1273 | Butanoic acid, 2-methylbutyl ester | E16 | - | - | - | - | - | - | - | - | - | 5.13^e^ | 20.81^a^ | - | 7.41^e^ | 12.2^c^ | - | 25^b^ | 15.03^c^ | - |
| 23.68 | 1533 | Heptanoic acid, butyl ester | E17 | - | - | - | - | - | - | - | - | - | - | 27.44^b^ | - | - | 22.77^b^ | - | - | 48.25^a^ | 48.39^a^ |
| 18.47 | 1320 | Hexanoic acid, propyl ester | E18 | - | - | - | - | - | - | - | - | - | - | 24.23^b^ | - | - | - | - | - | 27.03^b^ | 52.73^a^ |
| 21.15 | 1419 | Hexanoic acid, butyl ester | E19 | - | - | - | - | - | - | - | 16.12^f^ | - | 8.12^g^ | 253.68^b^ | 26.73^e^ | 7.46^g^ | 152.76^c^ | 243.35^b^ | 59.86^d^ | 241.91^b^ | 919.37^a^ |
| 26.17 | 1618 | Hexanoic acid, hexyl ester | E20 | - | - | - | - | - | - | - | - | - | 9.34^g^ | 148.42^d^ | 46.12^f^ | 7.36^g^ | 104.45^e^ | 828.48^b^ | 96.32^e^ | 174.13^c^ | 1879.67^a^ |
| 23.72 | 1508 | Hexanoic acid, pentyl ester | E21 | - | - | - | - | - | - | - | - | - | - | - | - | - | - | 41.72^b^ | 21.4^c^ | - | 115.38^a^ |
| 30.17 | 1802 | Octanoic acid, hexyl ester | E22 | - | - | - | - | - | - | - | - | - | - | - | - | - | - | 29.03^b^ | - | - | 56.24^a^ |
| 27.25 | 1678 | 2-Methylbutyl octanoate | E23 | - | - | - | - | - | - | - | - | - | - | - | - | - | 12.7 | - | - | 15.4 | 62.89 |
| 26.23 | 1614 | Butyl caprylate | E24 | - | - | - | - | - | - | - | - | - | - | 27.85^d^ | - | - | 38.24^c^ | 67.09^b^ | - | - | 185.72^a^ |
| 8.85 | 945 | Acetic acid propyl ester | E25 | - | - | - | - | - | - | - | - | - | - | - | - | - | - | - | - | 59.81 | - |
| 11.42 | 1069 | Acetic acid, butyl ester | E26 | - | - | - | - | - | - | - | 43.41^e^ | - | - | 252.98^b^ | - | - | 134.41^c^ | 99.23^d^ | - | 15.97f | 287.65^a^ |
| 17.18 | 1272 | Acetic acid, hexyl ester | E27 | 2.63^j^ | 14.73^h^ | 9.89^i^ | 4.93^j^ | - | 20.51^h^ | 5.24^j^ | 80.87^f^ | 36.99^g^ | 18.41^h^ | 539.6^c^ | 193.15^e^ | 15.64^h^ | 293.17^d^ | 836.12^b^ | - | 289.15^d^ | 2005.71^a^ |
| 14.27 | 1172 | Acetic acid, pentyl ester | E28 | - | - | - | - | - | - | - | 17.08^e^ | - | - | 38.33^c^ | - | - | 24.71^d^ | 31.81^c^ | - | 542.92^a^ | 46.79^b^ |
| 19.17 | 1346 | Propanoic acid, 2-methyl-, hexyl ester | E29 | - | - | - | - | - | - | - | - | - | - | - | - | - | 14.47^c^ | 38.79^b^ | - | 38.7^b^ | 117.82^a^ |
| 18.5 | 1248 | Propanoic acid, 2-methyl-, pentyl ester | E30 | - | - | - | - | - | - | - | - | - | - | - | - | - | - | - | 41.91 | - | - |
| 21.14 | 1432 | Butanoic acid, 2-methyl-, hexyl ester | E31 | - | - | - | - | - | - | 4.4^i^ | 54.57^h^ | - | 189.89^f^ | 851.81^c^ | 98.27^g^ | 198.28^f^ | 534.46^e^ | 1495.11^b^ | 543.73^e^ | 668.68d | 3898.95a |
| 16.58 | 1250 | 2-Methylbut-2-en-1-yl acetate | E32 | - | - | - | - | 112.36 | - | - | - | - | - | - | - | - | - | - | - | - | - |
| 18.57 | 1306 | 3-Hexen-1-ol, acetate, (E)- | E33 | - | - | - | - | 46.9 | - | - | - | - | - | - | - | - | - | - | - | - | - |
| 19.01 | 1321 | 2-Hexen-1-ol, acetate, (Z)- | E34 | - | - | - | - | 16.15 | - | - | - | - | - | - | - | - | - | - | - | - | - |
| 30.05 | 1798 | Methyl salicylate | E35 | 2.46^b^ | - | 9.19^a^ | - | - | - | - | - | - | - | - | - | - | - | - | - | - | - |
|  |  | **Sum** |  | 5.09^j^ | 30.65^g^ | 19.08^h^ | 4.93^j^ | 287.77^f^ | 35.73^g^ | 9.64^i^ | 785.99^e^ | 36.99^g^ | 333.33^f^ | 4679.68^b^ | 477.54^f^ | 386.84^f^ | 2653.09^c^ | 4554.62^b^ | 1206.15^d^ | 3775.7^c^ | 11628.43^a^ |
|  |  | **Aldehydes** |  |  |  |  |  |  |  |  |  |  |  |  |  |  |  |  |  |  |  |
| 11.78 | 1084 | Hexanal |  | 80.01^e^ | 330.68^a^ | 99.87^d^ | 78.34^e^ | 221.49^b^ | 237.22^b^ | 91.82^d^ | 148.05^c^ | 76.1^f^ | 125.71^c^ | 244.82^b^ | 45.83^g^ | 305.56^a^ | 122.28^c^ | 49.26^g^ | 140.9^c^ | 112.47^d^ | 95.79^d^ |
| 13.45 | 1142 | 4-Pentenal, 2-methyl- |  | - | - | - | - | 10.26 | - | - | - | - | - | - | - | - | - |  | - | - | - |
| 15.88 | 1225 | 2-Hexenal |  | 650.41^e^ | 496.02^e^ | 1170.1^c^ | 622.57^e^ | 348.2^f^ | 1481.61^a^ | 574.77^e^ | 297.15^f^ | 853.07^d^ | 1352.85^b^ | 1598.31^a^ | 1148.51^c^ | 1368.84^a^ | 893.72^d^ | 682.13^e^ | 1084.64^c^ | 821.38^d^ | 1052.13^c^ |
| 13.66 | 1146 | 3-Hexenal |  | 4.8^c^ | 9.94^b^ | - | 3.52^c^ | 13.64^a^ | - | - | 8.44^b^ | - | - | - | - | - | - | - | - | - | - |
| 17.18 | 1277 | Octanal |  | - | - | - | - | - | - | 3.89^c^ | - | - | - | - | - | - | 12.85^b^ | - | 13.59^b^ | 16.24^a^ | - |
| 18.9 | 1339 | 2-Heptenal, (Z)- |  | 3.13^d^ | - | - | 3.88^d^ | - | 9.17^c^ | 4.43^d^ | - | 12.48^c^ | 7.68^d^ | - | - | 8.61^d^ | 9.84^c^ | 25.93^b^ | 23.54^b^ | 13.28c | 44.32^a^ |
| 20.72 | 1388 | Nonanal |  | - | - | - | 2.78^d^ | 7.84^c^ | - | 5.96^c^ | 7.97^c^ | - | - | 36.57^a^ | - | - | 29.65^a^ | - | 22.31^b^ | 34.47^a^ | - |
| 21.79 | 1452 | 2-Octenal, (E)- |  | - | - | - | - | - | - | 3.79^c^ | - | - | - | - | - | 3.64^c^ | - | - | 13.78^a^ | 9.63^b^ | - |
| 21.17 | 1411 | 2,4-Hexadienal, (E,E)- |  | 2.87^b^ | 11.3^a^ | - | 4.35^b^ | 10.92^a^ | - | 2.8^b^ | - | - | - | - | - | - | - |  | - | - | - |
|  |  | **Sum** |  | 741.22^d^ | 847.94^d^ | 1269.97^b^ | 715.45^d^ | 612.33^d^ | 1728.01^a^ | 687.45^d^ | 461.62^e^ | 941.65^c^ | 1486.24^b^ | 1879.71^a^ | 1194.34^c^ | 1686.66^a^ | 1068.35^c^ | 757.33^d^ | 1298.75^b^ | 1007.48^c^ | 1192.24^c^ |
|  |  | **Alcohols** |  |  |  |  |  |  |  |  |  |  |  |  |  |  |  |  |  |  |  |
| 13.83 | 1146 | 1-Butanol |  | - | - | - | - | - | - | - | - | - | 7.86^b^ | - | - | 9.2^b^ | - | - | - | 53.29^a^ | - |
| 15.28 | 1208 | 1-Butanol, 2-methyl- |  | - | - | - | - | 7.94^e^ | - | 3.18^f^ | 22.37^d^ | - | 51.14^c^ | 75.52^b^ | - | 55.71^c^ | 39.23^d^ | - | 128.38a | 52.98c | - |
| 19.45 | 1387 | 1-Hexanol |  | - | - | - | - | - | - | 2.58^f^ | 11.73^e^ | - | 29.38^d^ | 75.48^b^ | 26.53^d^ | 30.27^d^ | 34.37^d^ | 50.3^c^ | 46.98^c^ | 75.62^b^ | 115.62a |
| 24.56 | 1582 | Linalool |  | 23.76^b^ | - | 32.56^a^ | - | - | - | - | - | - | - | - | - | - | - | - | - | - | - |
| 32.69 | 1896 | (5-Methyltetrahydro-2-Furanyl)Methanol |  | - | - | - | - | - | - | 3.85 | - | - | - | - | - | - | - | - | - | - | - |
|  |  | **Sum** |  | 23.76^e^ | - | 32.56^e^ | - | 7.94^f^ | - | 9.61^f^ | 34.1^e^ | - | 88.38^c^ | 151^b^ | 26.53^e^ | 95.18^c^ | 73.6^c^ | 50.3d | 175.36a | 181.89 | 115.62 |
|  |  | **Terpenoids** |  |  |  |  |  |  |  |  |  |  |  |  |  |  |  |  |  |  |  |
| 18.18 | 1305 | 1-Octen-3-one |  | - | - | - | - | - | - | - | - | - | - | - | - | - | - | - | 13.62^a^ | 14.84^a^ | - |
| 19.15 | 1338 | 5-Hepten-2-one, 6-methyl- |  | - | - | - | - | - | - | - | - | - | - | 19.92 | - | 5.58 | - | - | 19.1 | 17.14 | - |
| 29.11 | 1737 | (Z,E)-α-Farnesene |  | - | - | - | - | - | - | - | 8.63^g^ | - | 286.42^d^ | 1315.14^c^ | 29.28^f^ | 214.91^e^ | 332.1^d^ | 27.66^f^ | 1294.29^c^ | 1678.49^a^ | 14819.16^b^ |
| 29.82 | 1742 | Isocaryophillene |  | - | - | - | - | - | - | - | - | - | - | - | - | - | - | - | - | - | 40.58 |
|  |  | **Sum** |  | - | - | - | - | - | - | - | 8.63^g^ | - | 286.42^d^ | 1335.05^c^ | 29.28^f^ | 220.48^e^ | 332.1^d^ | 27.66^f^ | 1327.01^c^ | 1710.48^b^ | 14859.74^a^ |
|  |  | **Acids** |  |  |  |  |  |  |  |  |  |  |  |  |  |  |  |  |  |  |  |
| 17.45 | 1281 | Butanoic acid, 2-methyl- | - | - | - | - | - | - | - | - | 29.82^c^ | - | 10.53^d^ | 107.34^a^ | - | 11.92^d^ | 24.01^c^ | - | 25.79^c^ | 62.91^b^ | 49.13^b^ |

**Table S2**

| Sample | Library | Raw_reads | Clean_reads | Clean_bases | Error_rate | Q20 | Q30 | GC_pct |
| --- | --- | --- | --- | --- | --- | --- | --- | --- |
| F1_1 | FRAS192020449-1a | 47127348 | 45970394 | 6.9G | 0.03 | 96.88 | 91.49 | 47.33 |
| F1_2 | FRAS192020449-2a | 42957236 | 41620890 | 6.24G | 0.03 | 96.85 | 91.42 | 47.52 |
| F1_3 | FRAS192020449-3a | 45904192 | 44377528 | 6.66G | 0.03 | 96.68 | 91.28 | 47.09 |
| F2_1 | FRAS192020450-1a | 44320064 | 43106816 | 6.47G | 0.03 | 96.6 | 91.11 | 47.14 |
| F2_2 | FRAS192020450-2a | 56438242 | 55031026 | 8.25G | 0.03 | 96.73 | 91.15 | 47.48 |
| F2_3 | FRAS192020450-3a | 57345502 | 56144754 | 8.42G | 0.03 | 96.75 | 91.29 | 47.45 |
| F3_1 | FRAS192020451-1a | 46747178 | 45237016 | 6.79G | 0.03 | 96.95 | 91.83 | 47.45 |
| F3_2 | FRAS192020451-2a | 73873384 | 72359670 | 10.85G | 0.03 | 97.03 | 91.85 | 47.49 |
| F3_3 | FRAS192020451-3a | 47126128 | 45800758 | 6.87G | 0.03 | 96.89 | 91.72 | 47.36 |
| F4_1 | FRAS192020452-1a | 41194686 | 39883488 | 5.98G | 0.03 | 96.73 | 91.35 | 46.92 |
| F4_2 | FRAS192020452-2a | 59937112 | 58776666 | 8.82G | 0.03 | 96.66 | 91.04 | 47.04 |
| F4_3 | FRAS192020452-3a | 71497698 | 70208244 | 10.53G | 0.03 | 97.06 | 91.9 | 47.19 |
| F5_1 | FRAS192020453-1a | 60946908 | 59520250 | 8.93G | 0.03 | 97.19 | 92.19 | 47.03 |
| F5_2 | FRAS192020453-2a | 69326632 | 67614754 | 10.14G | 0.03 | 96.76 | 91.21 | 47.18 |
| F5_3 | FRAS192020453-3a | 66006160 | 64678836 | 9.7G | 0.03 | 96.9 | 91.5 | 47.13 |
| F6_1 | FRAS192020454-1a | 57172524 | 56203834 | 8.43G | 0.03 | 96.74 | 91.17 | 47.32 |
| F6_2 | FRAS192020454-2a | 55681696 | 54539516 | 8.18G | 0.03 | 96.83 | 91.3 | 47.22 |
| F6_3 | FRAS192020454-3a | 59851124 | 58646578 | 8.8G | 0.03 | 96.71 | 91.16 | 47.21 |
| P1_1 | FRAS192020455-1a | 56914650 | 55854384 | 8.38G | 0.03 | 96.82 | 91.36 | 46.98 |
| P1_2 | FRAS192020455-2a | 53966756 | 52843438 | 7.93G | 0.03 | 96.86 | 91.45 | 47.18 |
| P1_3 | FRAS192020455-3a | 58255266 | 57061414 | 8.56G | 0.03 | 96.89 | 91.49 | 47.12 |
| P2_1 | FRAS192020456-1a | 64772180 | 63511382 | 9.53G | 0.03 | 96.87 | 91.45 | 47.29 |
| P2_2 | FRAS192020456-2a | 57836534 | 56680772 | 8.5G | 0.03 | 96.63 | 90.98 | 47.33 |
| P2_3 | FRAS192020456-3a | 59210360 | 58208498 | 8.73G | 0.03 | 95.89 | 89.5 | 47.31 |
| P3_1 | FRAS192020457-1a | 54478668 | 53495302 | 8.02G | 0.03 | 97.06 | 91.95 | 47.37 |
| P3_2 | FRAS192020457-2a | 53038020 | 51786596 | 7.77G | 0.03 | 97.07 | 91.95 | 47.38 |
| P3_3 | FRAS192020457-3a | 62758870 | 61695120 | 9.25G | 0.03 | 96.92 | 91.62 | 47.15 |
| P4_1 | FRAS192020458-1a | 47459028 | 46641432 | 7.0G | 0.03 | 96.9 | 91.58 | 47.22 |
| P4_2 | FRAS192020458-2a | 63279202 | 61921390 | 9.29G | 0.03 | 97 | 91.8 | 47.18 |
| P4_3 | FRAS192020458-3a | 53844480 | 52637396 | 7.9G | 0.03 | 96.66 | 91.06 | 47.21 |
| P5_1 | FRAS192020459-1a | 61677066 | 60412122 | 9.06G | 0.03 | 97.08 | 91.97 | 47.4 |
| P5_2 | FRAS192020459-2a | 59000976 | 57796166 | 8.67G | 0.03 | 97.2 | 92.22 | 47.48 |
| P5_3 | FRAS192020459-3a | 58456302 | 57259400 | 8.59G | 0.03 | 97.07 | 91.92 | 47.48 |
| P6_1 | FRAS192020460-1a | 51140542 | 50167178 | 7.53G | 0.03 | 97.02 | 91.75 | 47.39 |
| P6_2 | FRAS192020460-2a | 60218882 | 58935152 | 8.84G | 0.03 | 97.3 | 92.42 | 47.39 |
| P6_3 | FRAS192020460-3a | 59925378 | 58723974 | 8.81G | 0.03 | 97.11 | 92.03 | 47.32 |
| X1_1 | FRAS192020461-1a | 68921778 | 67638296 | 10.15G | 0.03 | 97.07 | 91.96 | 47.41 |
| X1_2 | FRAS192020461-2a | 56233484 | 55092680 | 8.26G | 0.03 | 97.15 | 92.11 | 47.41 |
| X1_3 | FRAS192020461-3a | 54251260 | 53438938 | 8.02G | 0.03 | 96.97 | 91.75 | 47.28 |
| X2_1 | FRAS192020462-1a | 76656718 | 75470574 | 11.32G | 0.03 | 97.07 | 91.96 | 47.52 |
| X2_2 | FRAS192020462-2a | 51146176 | 50354694 | 7.55G | 0.03 | 96.76 | 91.15 | 47.61 |
| X2_3 | FRAS192020462-3a | 61539858 | 60431848 | 9.06G | 0.03 | 97.1 | 91.96 | 47.44 |
| X3_1 | FRAS192020463-1a | 63291278 | 62102870 | 9.32G | 0.03 | 96.9 | 91.55 | 47.19 |
| X3_2 | FRAS192020463-2a | 69127394 | 67804778 | 10.17G | 0.03 | 96.88 | 91.54 | 47.35 |
| X3_3 | FRAS192020463-3a | 59125580 | 58039810 | 8.71G | 0.03 | 96.95 | 91.69 | 47.24 |
| X4_1 | FRAS192020464-1a | 67795458 | 66461714 | 9.97G | 0.03 | 97.47 | 92.9 | 47.22 |
| X4_2 | FRAS192020464-2a | 58826172 | 57873276 | 8.68G | 0.03 | 96.97 | 91.69 | 47.25 |
| X4_3 | FRAS192020464-3a | 69119584 | 68078020 | 10.21G | 0.03 | 97.13 | 92.05 | 47.22 |
| X5_1 | FRAS192020465-1a | 61852880 | 60831588 | 9.12G | 0.03 | 96.82 | 91.43 | 47.34 |
| X5_2 | FRAS192020465-2a | 54458124 | 53597442 | 8.04G | 0.03 | 97.08 | 91.99 | 47.37 |
| X5_3 | FRAS192020465-3a | 58775132 | 57619762 | 8.64G | 0.03 | 97.08 | 91.95 | 47.3 |
| X6_1 | FRAS192020466-1a | 57096030 | 56125304 | 8.42G | 0.03 | 96.98 | 91.73 | 47.2 |
| X6_2 | FRAS192020466-2a | 55107020 | 54114656 | 8.12G | 0.03 | 97.19 | 92.16 | 47.03 |
| X6_3 | FRAS192020466-3a | 49064188 | 48222456 | 7.23G | 0.03 | 97.18 | 92.13 | 47.12 |

**Table S3**

| gene | F1 | | P1 | | X1 | | F2 | | P2 | | X2 | | F3 | | P3 | | X3 | | F4 | | P4 | | X4 | | F5 | | P5 | | X5 | | F6 | |
| --- | --- | --- | --- | --- | --- | --- | --- | --- | --- | --- | --- | --- | --- | --- | --- | --- | --- | --- | --- | --- | --- | --- | --- | --- | --- | --- | --- | --- | --- | --- | --- | --- |
| ACC-1 | 76.20 | d | 35.68 | f | 39.63 | f | 51.97 | e | 31.79 | f | 43.28 | f | 99.27 | c | 39.55 | f | 75.46 | d | 156.90 | b | 103.51 | c | 146.84 | b | 153.13 | b | 103.51 | c | 127.85 | c | 152.59 | b |
| ACC-2 | 38.49 | b | 23.75 | d | 27.21 | c | 24.04 | c | 22.89 | d | 25.14 | c | 43.37 | b | 18.75 | d | 31.96 | c | 49.91 | a | 31.77 | c | 42.29 | b | 44.96 | b | 31.77 | c | 37.79 | b | 51.40 | a |
| ACC-3 | 28.91 | c | 33.98 | b | 28.19 | c | 27.48 | c | 38.06 | b | 27.94 | c | 36.81 | b | 34.53 | b | 36.62 | b | 37.95 | b | 53.35 | b | 48.57 | b | 41.52 | b | 53.35 | b | 47.55 | b | 43.74 | b |
| MAT-1 | 31.63 | b | 14.49 | d | 19.07 | c | 19.21 | c | 14.22 | d | 16.20 | d | 27.34 | b | 12.75 | d | 21.68 | c | 50.04 | a | 40.41 | b | 54.77 | a | 47.03 | a | 35.59 | b | 48.18 | a | 47.17 | a |
| M1T-2 | 46.50 | e | 29.78 | f | 32.74 | e | 41.28 | e | 34.22 | e | 32.25 | e | 57.31 | d | 36.20 | e | 39.21 | b | 78.65 | b | 67.73 | b | 68.19 | b | 69.88 | b | 64.54 | b | 66.07 | b | 74.93 | b |
| KARIII | 22.43 | b | 25.42 | b | 24.91 | b | 18.10 | b | 20.15 | b | 18.82 | b | 21.34 | b | 19.79 | b | 21.27 | b | 37.40 | a | 28.86 | b | 34.77 | a | 36.41 | a | 37.84 | a | 34.21 | a | 37.77 | a |
| DH-1 | 3.91 | b | 0.56 | c | 0.61 | c | 4.44 | a | 0.60 | c | 0.30 | e | 4.70 | a | 0.53 | d | 0.67 | d | 4.78 | a | 0.67 | d | 0.59 | d | 2.74 | c | 0.56 | d | 0.45 | d | 2.45 | c |
| DH-2 | 53.38 | c | 40.32 | c | 48.54 | c | 48.56 | c | 44.53 | b | 65.94 | c | 92.27 | b | 55.57 | c | 91.72 | b | 110.80 | a | 87.90 | b | 115.13 | a | 90.49 | b | 91.89 | b | 97.68 | b | 103.09 | a |
| ER-1 | 21.64 | a | 14.99 | b | 12.41 | b | 18.53 | a | 15.63 | b | 15.49 | b | 15.00 | b | 16.58 | b | 15.67 | b | 13.71 | b | 18.40 | a | 14.52 | b | 10.24 | b | 14.73 | c | 9.98 | c | 9.61 | c |
| ER-2 | 59.87 | e | 20.79 | c | 36.09 | c | 32.24 | c | 19.46 | c | 29.40 | c | 81.23 | d | 21.56 | c | 46.71 | e | 147.48 | b | 87.33 | d | 125.58 | c | 125.36 | c | 14.73 | g | 117.51 | c | 148.90 | b |
| ER-3 | 11.42 | d | 16.78 | b | 22.26 | b | 6.57 | e | 13.74 | d | 15.80 | d | 11.68 | d | 14.67 | d | 24.28 | b | 21.39 | b | 36.49 | b | 43.73 | b | 21.00 | b | 14.73 | d | 42.96 | b | 23.31 | b |
| KARII-1 | 22.44 | b | 13.87 | c | 20.16 | b | 21.15 | b | 11.53 | c | 21.55 | b | 20.57 | b | 16.21 | c | 24.80 | b | 37.01 | a | 26.62 | b | 38.64 | a | 37.75 | a | 14.73 | c | 34.37 | a | 39.48 | a |
| KARII-2 | 7.39 | c | 3.59 | c | 4.97 | c | 7.60 | c | 2.44 | d | 4.77 | c | 7.02 | c | 3.22 | c | 5.76 | c | 17.35 | a | 6.19 | c | 12.82 | b | 18.91 | a | 14.73 | b | 10.98 | b | 18.09 | a |
| SAD | 39.56 | b | 38.56 | b | 21.51 | c | 33.25 | b | 35.87 | b | 20.48 | c | 45.74 | a | 33.75 | b | 25.79 | c | 49.96 | a | 48.32 | a | 27.03 | c | 53.45 | a | 14.73 | d | 32.29 | b | 47.12 | a |
| OTE | 12.68 | a | 10.78 | b | 11.14 | b | 9.57 | b | 9.48 | b | 8.28 | c | 10.22 | b | 9.36 | b | 9.24 | b | 15.17 | a | 11.15 | b | 13.13 | a | 15.64 | a | 11.88 | b | 11.67 | b | 14.46 | a |
| FAD-1 | 141.42 | e | 190.61 | e | 293.78 | d | 299.55 | d | 367.58 | d | 731.80 | b | 338.63 | d | 512.87 | c | 621.40 | b | 296.42 | d | 602.45 | b | 488.58 | c | 320.66 | d | 587.11 | b | 451.55 | c | 341.85 | d |
| FAD-2 | 70.47 | a | 58.28 | b | 58.92 | b | 37.61 | b | 47.41 | b | 46.57 | b | 21.50 | d | 29.46 | c | 23.79 | c | 17.63 | d | 22.20 | c | 31.28 | c | 20.01 | c | 41.49 | b | 34.65 | c | 42.58 | b |
| LOX-1 | 198.59 | d | 54.44 | e | 193.92 | d | 294.67 | c | 54.44 | e | 160.49 | d | 344.37 | c | 51.26 | e | 274.58 | c | 785.50 | b | 204.27 | d | 699.98 | b | 741.95 | b | 204.27 | d | 360.21 | c | 1003.59 | a |
| LOX-2 | 13.92 | g | 4.59 | h | 10.24 | g | 116.07 | e | 4.59 | h | 14.66 | g | 373.15 | c | 6.90 | g | 84.47 | e | 566.42 | b | 32.65 | f | 342.78 | c | 476.12 | b | 32.65 | f | 247.03 | c | 981.63 | a |
| LOX-3 | 31.25 | b | 0.77 | d | 6.78 | c | 48.36 | b | 0.77 | d | 5.25 | c | 58.59 | b | 1.25 | d | 8.94 | c | 146.44 | a | 3.34 | c | 26.51 | b | 144.24 | a | 3.34 | c | 10.43 | c | 178.95 | a |
| LOX-4 | 0.04 | e | 0.08 | e | 0.02 | e | 0.05 | e | 0.08 | e | 0.00 | f | 0.24 | d | 0.05 | e | 0.05 | e | 8.16 | b | 0.28 | d | 2.94 | c | 15.79 | a | 0.27 | d | 5.81 | b | 18.59 | a |
| LOX-5 | 29.32 | a | 21.70 | a | 19.42 | a | 19.88 | a | 21.70 | a | 28.39 | a | 14.68 | b | 15.15 | b | 27.64 | a | 6.51 | c | 6.63 | c | 6.14 | c | 3.24 | d | 6.63 | c | 7.40 | c | 2.28 | d |
| LOX-6 | 4.63 | b | 5.84 | a | 2.97 | b | 3.96 | b | 5.74 | a | 4.00 | b | 1.71 | c | 3.59 | b | 3.22 | b | 0.50 | d | 0.97 | c | 0.63 | d | 0.37 | d | 1.09 | c | 0.35 | d | 0.17 | d |
| HPL | 259.26 | a | 316.40 | a | 283.75 | a | 289.44 | a | 323.45 | a | 276.47 | a | 190.77 | b | 233.11 | a | 224.38 | a | 172.63 | b | 217.44 | a | 155.02 | b | 116.59 | b | 143.84 | b | 126.28 | b | 126.43 | b |
| ADH-1 | 29.25 | a | 31.19 | a | 28.73 | a | 27.01 | a | 32.47 | a | 25.59 | a | 13.27 | b | 17.91 | b | 12.63 | b | 9.82 | b | 12.15 | b | 6.93 | c | 7.71 | c | 9.63 | c | 7.25 | c | 7.14 | c |
| ADH-2 | 38.39 | b | 25.34 | b | 27.11 | b | 48.05 | a | 29.97 | b | 31.94 | b | 31.44 | b | 28.28 | b | 30.98 | b | 30.36 | b | 29.88 | b | 27.83 | b | 24.44 | b | 20.98 | b | 27.58 | b | 22.60 | c |
| ADH-3 | 27.14 | b | 18.62 | b | 12.98 | c | 45.98 | a | 30.62 | b | 28.79 | b | 55.54 | a | 28.36 | b | 13.98 | c | 17.75 | b | 25.73 | b | 11.85 | c | 12.07 | c | 20.56 | b | 16.37 | b | 21.01 | b |
| AAT-1 | 48.39 | c | 38.66 | c | 58.18 | c | 26.96 | d | 26.98 | d | 21.72 | d | 36.39 | c | 33.44 | c | 39.20 | c | 76.93 | b | 57.41 | c | 37.39 | c | 165.14 | a | 65.40 | b | 88.02 | b | 90.31 | b |
| AAT-2 | 49.59 | c | 38.00 | d | 40.57 | d | 36.27 | d | 30.88 | d | 19.17 | e | 59.71 | c | 37.89 | d | 34.70 | d | 133.56 | b | 88.91 | c | 39.00 | d | 202.40 | a | 120.95 | b | 76.04 | c | 137.39 | b |
| AAT-3 | 23.60 | c | 17.76 | d | 18.45 | d | 15.75 | d | 11.73 | d | 7.17 | d | 17.89 | d | 15.68 | d | 11.78 | d | 67.67 | b | 43.61 | b | 37.15 | c | 125.04 | a | 89.94 | b | 59.62 | c | 92.95 | a |
| TS | 14.08 | b | 8.39 | c | 10.68 | c | 16.60 | b | 7.89 | c | 12.64 | b | 16.00 | b | 9.49 | c | 14.53 | b | 35.67 | a | 21.53 | b | 33.20 | a | 38.59 | a | 21.33 | b | 35.24 | a | 37.57 | a |
| TD | 33.27 | b | 40.65 | b | 36.29 | b | 51.48 | a | 32.68 | b | 35.37 | b | 44.28 | a | 33.89 | b | 36.13 | b | 48.32 | a | 31.03 | b | 47.41 | a | 53.88 | a | 38.84 | b | 49.74 | a | 48.19 | a |
| AHIR-1 | 107.20 | a | 109.80 | a | 98.06 | a | 119.75 | a | 99.42 | a | 109.44 | a | 80.15 | b | 96.71 | a | 85.46 | b | 90.60 | b | 83.13 | b | 76.53 | c | 72.70 | c | 76.59 | c | 59.61 | d | 86.08 | b |
| AHIR-2 | 1.51 | c | 48.34 | a | 25.67 | b | 2.26 | c | 45.10 | a | 25.42 | b | 2.38 | c | 43.34 | a | 23.83 | b | 4.64 | c | 45.08 | a | 25.82 | b | 3.78 | c | 41.33 | a | 23.45 | b | 5.06 | c |
| DHAD | 25.90 | a | 31.41 | a | 29.50 | a | 26.88 | a | 23.47 | a | 30.98 | a | 17.17 | b | 24.37 | a | 25.47 | a | 15.00 | b | 18.24 | b | 21.43 | a | 13.78 | b | 18.31 | b | 16.75 | b | 12.86 | b |
| BCAT-1 | 8.01 | b | 7.36 | b | 7.14 | b | 9.04 | b | 9.11 | b | 5.40 | c | 13.72 | b | 11.82 | b | 10.48 | b | 15.86 | a | 15.23 | a | 11.13 | b | 20.54 | a | 20.74 | a | 15.28 | a | 17.37 | a |
| BCAT-2 | 25.57 | a | 31.69 | a | 31.88 | a | 18.55 | a | 23.05 | a | 22.34 | b | 16.84 | b | 14.23 | b | 17.66 | b | 14.31 | b | 9.89 | c | 11.47 | c | 13.47 | c | 13.47 | b | 13.42 | b | 17.08 | b |
| PD-1 | 20.18 | f | 2.29 | g | 1.72 | g | 114.48 | d | 0.37 | h | 0.38 | h | 327.66 | c | 5.25 | g | 9.94 | g | 500.28 | b | 33.89 | e | 45.72 | e | 365.66 | c | 35.03 | e | 31.60 | e | 846.11 | a |
| PD-2 | 108.67 | b | 50.99 | c | 92.97 | b | 58.56 | c | 28.56 | d | 74.89 | c | 71.90 | c | 45.61 | c | 100.27 | b | 148.65 | b | 105.69 | b | 163.10 | b | 210.26 | a | 105.71 | b | 205.75 | a | 165.97 | a |
| ALDH-1 | 51.44 | c | 37.11 | c | 25.17 | c | 76.62 | b | 68.39 | b | 84.66 | b | 138.94 | a | 75.28 | b | 85.34 | b | 132.32 | a | 152.49 | a | 96.19 | b | 73.59 | b | 117.44 | b | 77.32 | b | 110.59 | b |
| ALDH-2 | 150.84 | c | 115.78 | c | 151.39 | c | 166.09 | c | 129.12 | c | 131.74 | c | 236.34 | b | 145.53 | c | 206.11 | b | 318.99 | a | 217.70 | b | 261.64 | b | 302.42 | a | 236.28 | b | 251.83 | b | 356.13 | a |
| ALDH-3 | 71.73 | c | 57.20 | c | 108.92 | b | 83.74 | c | 68.72 | c | 106.26 | b | 140.49 | b | 72.45 | c | 120.94 | b | 187.28 | a | 130.10 | b | 193.67 | a | 160.45 | a | 128.65 | b | 170.65 | a | 211.54 | a |
| CEX-1 | 45.95 | a | 4.89 | d | 22.12 | b | 37.46 | b | 5.01 | d | 25.93 | b | 63.80 | a | 5.14 | d | 8.98 | c | 62.70 | a | 3.09 | d | 12.64 | c | 69.16 | a | 12.03 | c | 19.30 | b | 74.34 | a |
| CEX-2 | 52.69 | b | 72.82 | b | 135.70 | a | 30.58 | c | 67.94 | b | 95.75 | a | 29.56 | c | 69.03 | b | 108.11 | a | 25.30 | c | 87.48 | b | 109.11 | a | 25.59 | c | 93.43 | b | 88.85 | b | 27.34 | c |
| CEX-3 | 6.78 | c | 7.02 | c | 5.18 | c | 6.05 | c | 9.53 | c | 6.62 | c | 7.25 | c | 6.32 | c | 7.17 | c | 20.41 | b | 21.89 | b | 24.14 | b | 17.57 | b | 30.55 | a | 35.73 | a | 20.63 | b |
| CEX-4 | 32.27 | c | 33.44 | c | 85.49 | a | 14.81 | d | 33.42 | c | 36.64 | c | 6.95 | d | 50.95 | b | 24.76 | c | 3.63 | d | 33.14 | c | 6.90 | d | 2.14 | e | 53.97 | b | 5.79 | d | 0.81 | f |
| ACOAAT-1 | 102.98 | d | 303.47 | a | 158.26 | b | 76.51 | e | 126.47 | c | 61.64 | f | 103.83 | d | 191.32 | b | 79.74 | e | 136.51 | b | 147.02 | b | 100.25 | d | 178.12 | b | 293.04 | a | 130.08 | c | 179.94 | b |
| ACOAAT-2 | 21.60 | a | 26.79 | a | 22.47 | a | 24.88 | a | 29.85 | a | 24.14 | a | 20.77 | a | 22.35 | a | 18.62 | a | 18.02 | a | 22.42 | a | 19.53 | a | 14.99 | b | 20.72 | a | 17.22 | a | 14.10 | b |
| ACOAAT-3 | 413.71 | a | 230.28 | d | 214.43 | d | 281.70 | c | 268.51 | c | 261.31 | c | 443.48 | a | 216.61 | d | 155.92 | f | 338.99 | c | 187.19 | e | 223.22 | d | 307.96 | c | 289.17 | c | 294.57 | c | 386.03 | b |
| HMGS-1 | 76.09 | f | 91.32 | e | 91.85 | e | 39.35 | h | 40.49 | h | 34.80 | h | 82.40 | f | 81.86 | f | 51.26 | g | 110.52 | d | 61.96 | g | 82.85 | f | 141.11 | c | 172.82 | b | 118.97 | d | 167.50 | b |
| HMGS-2 | 4.02 | b | 12.91 | a | 5.56 | b | 1.75 | c | 4.63 | b | 1.16 | d | 2.18 | c | 9.55 | a | 1.65 | c | 2.66 | c | 4.91 | b | 1.85 | c | 3.95 | b | 9.34 | a | 2.12 | c | 3.53 | b |
| HMGR-1 | 71.61 | b | 120.09 | a | 127.00 | a | 51.91 | c | 80.49 | b | 66.64 | b | 52.62 | c | 83.17 | b | 49.21 | c | 57.06 | c | 48.08 | d | 51.70 | c | 61.98 | c | 78.18 | b | 63.80 | c | 63.79 | c |
| HMGR-2 | 173.58 | c | 110.23 | e | 189.39 | c | 186.53 | c | 109.68 | e | 211.98 | c | 191.09 | c | 147.01 | d | 133.47 | d | 265.74 | b | 143.82 | d | 231.98 | c | 323.66 | a | 242.44 | c | 273.95 | b | 360.93 | a |
| MK-1 | 19.52 | c | 16.72 | d | 15.41 | d | 11.56 | d | 13.62 | d | 11.53 | d | 23.95 | c | 27.34 | c | 13.45 | d | 34.28 | b | 21.38 | c | 20.65 | c | 42.46 | b | 57.95 | a | 35.57 | b | 41.49 | b |
| MK-2 | 4.14 | c | 10.49 | a | 11.52 | a | 2.95 | d | 6.66 | b | 4.34 | c | 2.25 | d | 4.85 | c | 3.41 | c | 1.70 | d | 3.05 | c | 1.72 | d | 1.98 | d | 2.54 | d | 1.85 | d | 2.13 | d |
| PMK | 20.52 | b | 20.37 | b | 20.31 | b | 19.52 | b | 12.97 | c | 15.18 | c | 26.64 | b | 20.99 | b | 16.66 | c | 28.91 | a | 19.17 | b | 20.83 | b | 34.48 | a | 30.21 | a | 22.73 | b | 35.88 | a |
| M5DD-1 | 18.21 | e | 35.20 | d | 35.20 | d | 12.69 | f | 15.31 | f | 16.97 | f | 33.26 | d | 41.51 | c | 21.00 | e | 30.28 | d | 26.72 | d | 21.00 | e | 55.52 | b | 73.03 | a | 43.54 | c | 44.69 | c |
| M5DD-2 | 48.58 | b | 44.72 | b | 51.16 | b | 31.17 | c | 28.77 | c | 33.47 | c | 57.61 | b | 50.01 | b | 37.42 | c | 51.50 | b | 33.59 | c | 37.42 | c | 58.27 | b | 76.89 | a | 57.03 | b | 65.81 | b |
| IDDI-1 | 19.81 | c | 26.23 | c | 26.88 | c | 14.92 | d | 16.01 | d | 10.47 | d | 33.87 | c | 27.75 | c | 19.91 | d | 33.50 | c | 20.13 | d | 19.91 | d | 44.41 | a | 48.88 | a | 29.21 | c | 48.98 | a |
| IDDI-2 | 26.40 | e | 146.22 | b | 208.41 | a | 14.02 | f | 26.15 | e | 15.33 | f | 30.76 | e | 77.73 | c | 25.70 | e | 31.08 | e | 16.61 | f | 25.70 | e | 57.16 | d | 64.62 | c | 33.85 | e | 51.85 | d |
| FDPS-1 | 49.01 | d | 53.69 | d | 36.85 | e | 30.44 | e | 53.35 | d | 34.19 | e | 53.96 | d | 57.79 | d | 50.37 | d | 66.74 | d | 62.00 | d | 53.37 | d | 62.91 | d | 99.33 | b | 55.43 | d | 78.84 | c |
| FDPS-2 | 13.89 | d | 35.69 | a | 44.25 | a | 8.44 | d | 13.07 | d | 9.90 | d | 18.72 | c | 31.47 | a | 16.25 | c | 28.87 | b | 19.54 | c | 21.64 | b | 29.00 | b | 40.13 | a | 24.14 | b | 30.32 | a |
| FDPS-3 | 4.79 | g | 5.56 | g | 8.49 | f | 4.13 | g | 2.83 | g | 2.88 | g | 15.98 | e | 12.51 | e | 8.64 | f | 29.89 | d | 12.31 | e | 24.03 | d | 34.74 | d | 41.48 | c | 31.68 | d | 56.16 | b |
| FDPS-4 | 30.72 | c | 23.52 | d | 30.48 | c | 18.27 | d | 20.41 | d | 27.07 | d | 36.85 | b | 34.65 | b | 28.67 | c | 40.71 | b | 30.48 | c | 32.97 | c | 47.90 | b | 58.93 | a | 47.47 | b | 49.14 | a |
| AFS | 7.38 | i | 0.25 | k | 1.24 | j | 5.97 | i | 0.20 | k | 0.22 | k | 94.88 | h | 0.67 | i | 4.83 | k | 529.29 | d | 84.31 | h | 271.68 | f | 427.16 | e | 182.00 | g | 313.28 | f | 944.00 | b |
| MYC2-1 | 35.24 | e | 86.75 | a | 74.08 | a | 67.05 | b | 51.84 | c | 47.23 | c | 24.29 | f | 40.26 | d | 32.37 | e | 36.32 | e | 35.31 | e | 42.96 | d | 41.82 | d | 44.73 | d | 42.16 | d | 45.55 | d |
| MYC2-2 | 6.08 | b | 12.65 | a | 7.75 | b | 7.03 | b | 11.03 | a | 8.54 | b | 4.25 | c | 8.55 | b | 5.60 | c | 3.09 | d | 4.66 | c | 2.80 | d | 2.74 | d | 15.52 | a | 5.34 | c | 2.73 | d |
| ERF-1 | 4.06 | g | 6.84 | g | 5.41 | g | 4.36 | g | 4.36 | g | 2.47 | h | 11.08 | f | 10.57 | f | 5.19 | g | 63.76 | c | 9.44 | f | 28.30 | e | 121.36 | a | 28.14 | e | 43.96 | d | 121.57 | a |
| ERF-2 | 433.85 | b | 6.84 | f | 292.77 | e | 442.94 | b | 441.94 | b | 473.71 | b | 652.31 | a | 390.80 | c | 310.38 | d | 480.34 | b | 374.05 | c | 344.52 | d | 330.10 | d | 293.58 | e | 290.64 | e | 397.88 | c |
| ERF-3 | 118.37 | d | 6.84 | e | 170.01 | c | 156.29 | c | 156.29 | c | 191.36 | b | 150.21 | c | 168.86 | b | 211.85 | b | 152.48 | c | 219.00 | b | 212.44 | b | 157.04 | c | 279.80 | b | 249.85 | b | 164.91 | c |
| ERF-4 | 49.74 | g | 6.84 | h | 68.39 | f | 108.97 | e | 107.97 | e | 70.20 | f | 198.84 | c | 126.29 | d | 135.75 | d | 203.49 | c | 271.29 | b | 203.17 | c | 242.16 | b | 242.94 | b | 234.47 | b | 255.58 | b |
| WRKY-1 | 28.57 | e | 17.10 | f | 26.35 | e | 44.54 | c | 21.28 | f | 32.92 | d | 51.41 | c | 28.53 | e | 35.28 | d | 41.82 | d | 41.95 | d | 50.18 | c | 53.45 | c | 47.71 | c | 46.81 | c | 61.15 | b |
| WRKY-2 | 29.87 | c | 26.51 | c | 38.36 | b | 30.49 | c | 30.63 | c | 31.87 | c | 38.71 | b | 36.36 | b | 38.72 | b | 50.78 | a | 49.45 | a | 54.12 | a | 50.41 | a | 45.63 | b | 43.37 | b | 52.86 | a |
| MYB-1 | 40.37 | e | 76.61 | c | 99.78 | b | 31.13 | f | 91.52 | b | 57.31 | d | 58.31 | d | 88.37 | b | 75.84 | c | 55.70 | d | 105.01 | b | 91.27 | b | 67.55 | c | 125.23 | c | 114.84 | c | 58.28 | d |
| MYB-2 | 161.98 | a | 31.45 | f | 62.31 | d | 86.47 | c | 90.20 | c | 112.01 | b | 112.01 | b | 48.53 | b | 75.44 | d | 151.84 | b | 69.03 | e | 92.07 | d | 86.56 | a | 86.81 | d | 96.51 | c | 116.19 | b |
| MYB-3 | 63.26 | e | 76.27 | d | 105.82 | c | 46.80 | f | 95.70 | c | 50.32 | f | 50.32 | f | 38.23 | g | 81.36 | d | 67.74 | e | 95.75 | c | 99.77 | c | 76.39 | d | 64.45 | e | 109.43 | c | 91.39 | c |
| MYB-4 | 6.24 | e | 4.98 | f | 8.65 | e | 10.14 | e | 16.21 | d | 18.00 | d | 18.00 | d | 11.98 | e | 14.80 | d | 41.85 | b | 30.81 | c | 46.41 | b | 48.93 | b | 43.94 | b | 51.67 | b | 51.95 | b |
| BZIP-1 | 593.08 | e | 317.74 | i | 514.40 | f | 446.51 | g | 382.05 | h | 618.81 | e | 931.86 | a | 394.03 | h | 612.60 | e | 965.97 | a | 618.67 | e | 739.65 | c | 725.72 | c | 581.96 | e | 661.57 | d | 911.06 | a |
| BZIP-2 | 792.08 | g | 791.33 | g | 1106.16 | e | 301.07 | i | 919.82 | f | 652.77 | h | 1015.08 | e | 856.75 | f | 892.90 | f | 1271.49 | d | 1468.69 | c | 1313.85 | d | 1158.89 | e | 1519.64 | b | 1288.54 | d | 1330.96 | d |
| MADS-box-1 | 47.24 | d | 20.05 | f | 34.13 | e | 41.71 | e | 15.45 | g | 26.55 | f | 66.37 | c | 18.45 | f | 33.25 | e | 83.00 | b | 28.68 | f | 50.67 | d | 81.51 | b | 35.22 | e | 56.04 | d | 92.89 | a |
| MADS-box-2 | 79.76 | g | 80.64 | g | 100.60 | f | 70.40 | h | 82.86 | g | 94.71 | f | 100.37 | f | 94.04 | f | 104.18 | f | 124.76 | e | 180.40 | b | 131.56 | e | 112.66 | f | 203.76 | b | 153.74 | c | 142.44 | c |
| MADS-box-3 | 180.49 | b | 181.76 | b | 237.35 | a | 145.91 | d | 192.60 | b | 186.41 | b | 212.09 | b | 193.24 | b | 200.99 | b | 215.43 | b | 202.09 | b | 163.35 | c | 183.82 | b | 191.49 | b | 184.68 | b | 206.51 | b |
| MADS-box-4 | 253.87 | a | 218.47 | b | 237.21 | a | 148.64 | d | 271.66 | a | 187.41 | c | 81.00 | f | 204.14 | b | 152.43 | d | 69.65 | f | 188.88 | c | 112.12 | e | 73.63 | f | 133.12 | d | 125.93 | e | 61.15 | g |

**Table S4**

| Gene ID | Gene name | Forward primer sequence | Forward primer sequence |
| --- | --- | --- | --- |
| MD07G1004500 | LOX5a | CGAGACAGCACTTCTTGAT | TAGCCTTAGGTAACTTGACTC |
| MD09G1069500 | LOX1a | TCAACATTCGCCTTCTTCA | GTTAGTCCATCCAAGTTATTCC |
| MD02G1013900 | AAT-1 | ACTAGGAGACACAATTCTACC | GTCAGGAACAGGAGCAAT |
| MD05G1191100 | CXE-2 | GCAATATCTGTAAGGCTCTAC | GCAACAACATTGGCTTCA |
| MD04G1003600 | DHAD | CTATATTCCATCGCCATACAAG | CTCATCAGTCACGCATCC |
| MD10G1311000 | AFS | GAGGCAGAGTGGTATAATAAGA | TTGAGGCGAACGATGAGA |
| MD12G1057600 | FAD-1 | GTCTCAACCTCTATCCTTCTT | CAGTGTCATCCAGCCATT |
| MD13G1163300 | ERF-1 | GTGTGGTGGTGCTATCATT | GGTCAGAGATGGTGTCAAG |
| MD06G1217200 | MYB-1 | TTGATTCTTGACCTCCACTC | CCTTGTTCTCCAATAGTTCTTG |
| MD10G1324500 | WRKY-1 | TGAGAGTCCTGAATCTGAATC | ATGGTAGCCTCGGTTGAT |
| MD06G1204300 | MADS-box-2 | GCTTCCTTCCAACTCAGA | TCCATTCCATTATGTGAACC |
| MD04G1069200 | HMGS-1 | CTATCGCTGATGCTGAGTA | CTTACGGAGTCATTGAACAC |

**Fig. S1**


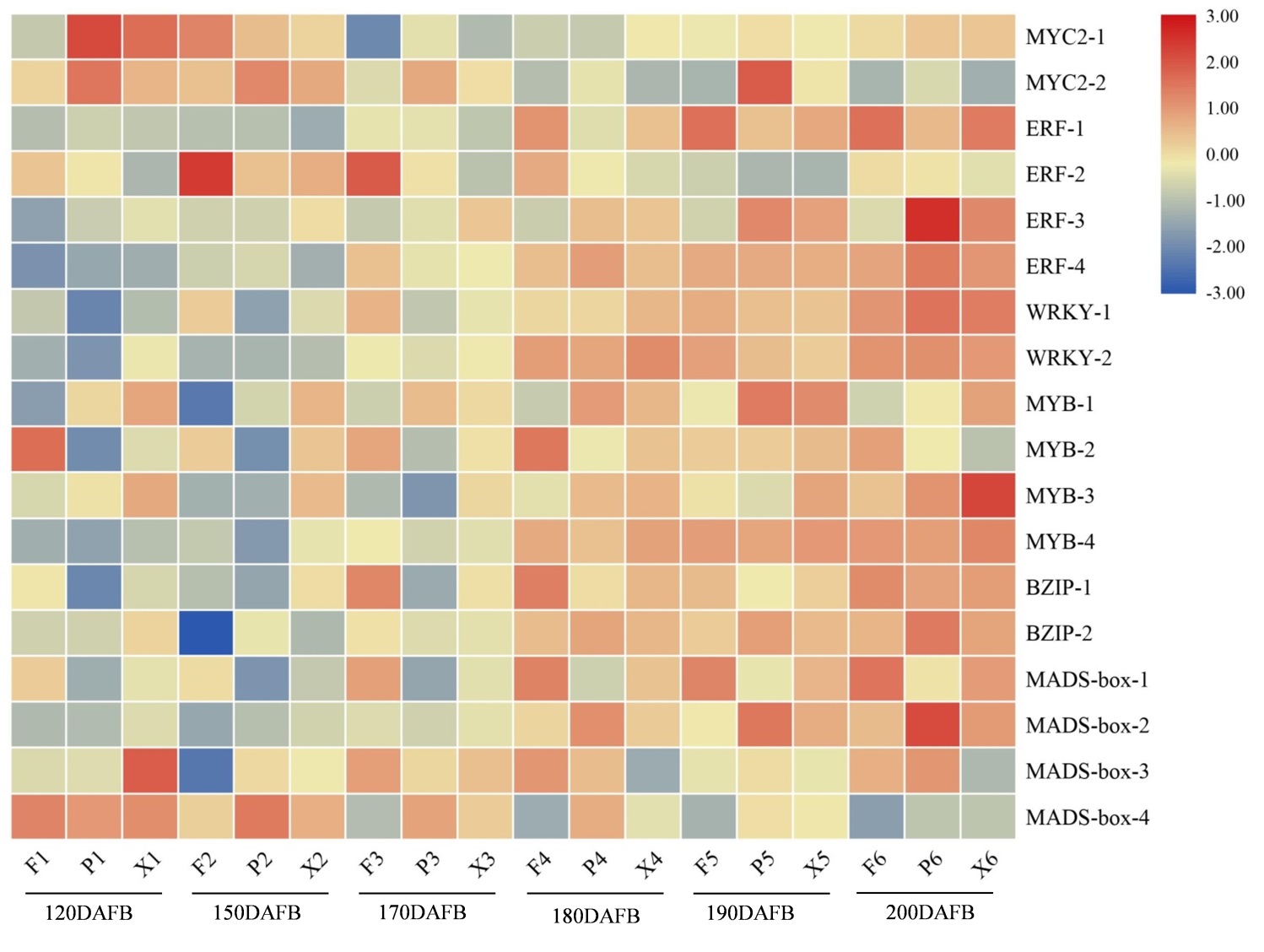

Supplement: Supplementary file 1 — Additional file 1: Supplemental Table S1. Changes in volatiles content of‘Ruixue’ and its parents apples during fruit development. “RX” represents ‘Ruixue’; “FJ” represents ‘Fuji’; “PL” represents ‘Pink Lady’. The six different fruit developmental stages of‘Ruixue’, ‘Pink Lady’ and ‘Fuji’, namely at 120, 150, 170, 180, 190 and 200 DAFB (days after full bloom ). “–” represents no detected. Means with different letters are significantly different at P <0.05, Duncan’s new multiple range test. Supplemental Table S2. Throughput and quality of RNA-seq in ‘Ruixue’ and its parents apples after filter. Supplemental Table S3. Analyzed expression level of volatile-related genes of the FPKM values in Ruixue’ and its parents apples during fruit development. “F1, F2 F3, F4, F5 and F6” respectively represents at 120, 150, 170, 180, 190 and 200 DAFB(days after full bloom ) of ‘Fuji’; “P1, P2, P3, P4, P5 and P6 ” respectively represents1 at 120, 150, 170, 180, 190 and 200 DAFB(days after full bloom ) of ‘Pink Lady’; “X1, X2, X3, X4, X5 and X6” respectively represents at 120 ,150,170,180,190 and 200 DAFB(days after full bloom ) of ‘Ruixue’. Means with different letters are significantly different at P <0.05, Duncan’s new multiple range test. Supplemental Table S4. Gene-specific primers used for RT-qPCR analysis. Supplemental Fig. S1. Analyzed expression patterns of transcription factors in Ruixue’ and its parents apples during fruit development by Heatmap. [file 12870_2021_3032_MOESM1_ESM.docx]
